# 8-Hydroxyquinoline-2-Carboxylic Acid as Possible Molybdophore: A Multi-Technique Approach to Define Its Chemical Speciation, Coordination and Sequestering Ability in Aqueous Solution

**DOI:** 10.3390/biom10060930

**Published:** 2020-06-18

**Authors:** Katia Arena, Giuseppe Brancato, Francesco Cacciola, Francesco Crea, Salvatore Cataldo, Concetta De Stefano, Sofia Gama, Gabriele Lando, Demetrio Milea, Luigi Mondello, Alberto Pettignano, Winfried Plass, Silvio Sammartano

**Affiliations:** 1 Dipartimento di Scienze Chimiche, Biologiche, Farmaceutiche ed Ambientali, Università degli Studi di Messina, Viale F. Stagno d’Alcontres 31, 98166 Messina, Italy; arenak@unime.it (K.A.); fcrea@unime.it (F.C.); cdestefano@unime.it (C.D.S.); glando@unime.it (G.L.); lmondello@unime.it (L.M.); ssammartano@unime.it (S.S.); 2 Scuola Normale Superiore, Palazzo della Carovana, Classe di Scienze Matematiche e Naturali, Piazza dei Cavalieri, 7, 56126 Pisa, Italy; giuseppe.brancato@sns.it; 3 Dipartimento di Scienze Biomediche, Odontoiatriche e delle Immagini Morfologiche e Funzionali, Università degli Studi di Messina, Viale Consolare Valeria s.n., 98125 Messina, Italy; cacciolaf@unime.it; 4 Dipartimento di Fisica e Chimica Emilio Segrè, Università degli Studi di Palermo, Viale delle Scienze, Ed. 17., 90128 Palermo, Italy; salvatore.cataldo@unipa.it (S.C.); alberto.pettignano@unipa.it (A.P.); 5 Department of Analytical Chemistry, Faculty of Chemistry, University of Białystok, ul. Ciołkowskiego 1K, 15-245 Białystok, Poland; 6 Chromaleont s.r.l., c/o Dipartimento di Scienze Chimiche, Biologiche, Farmaceutiche ed Ambientali, Università degli Studi di Messina, Viale Annunziata, 98168 Messina, Italy; 7 Facoltà Dipartimentale di Scienze e Tecnologie per l’Uomo e l’Ambiente, Università Campus Bio-Medico di Roma, 00128 Roma, Italy; 8 BeSep s.r.l., c/o Dipartimento di Scienze Chimiche, Biologiche, Farmaceutiche ed Ambientali, Università degli Studi di Messina, Viale Annunziata, 98168 Messina, Italy; 9 Institut für Anorganische und Analytische Chemie, Friedrich-Schiller-Universität Jena, Humboldtstr 8, 07743 Jena, Germany; sekr.plass@uni-jena.de

**Keywords:** chemical speciation, metal complexes, metallophores, molybdate, natural chelants, sequestration, stability constants

## Abstract

8-hydroxyquinoline-2-carboxylic acid (*8-HQA*) has been found in high concentrations (0.5–5.0 mmol·dm^−3^) in the gut of Noctuid larvae (and in a few other lepidopterans), in which it is proposed to act as a siderophore. Since it is known that many natural siderophores are also involved in the uptake and metabolism of other essential elements than iron, this study reports some results on the investigation of *8-HQA* interactions with molybdate (MoO_4_^2−^, i.e., the main molybdenum form in aqueous environments), in order to understand the possible role of this ligand as molybdophore. A multi-technique approach has been adopted, in order to derive a comprehensive set of information necessary to assess the chemical speciation of the *8-HQA*/MoO_4_^2−^ system, as well as the coordination behavior and the sequestering ability of *8-HQA* towards molybdate. Chemical speciation studies have been performed in KCl_(aq)_ at *I* = 0.2 mol·dm^−3^ and *T* = 298.15 K by ISE-H^+^ (glass electrode) potentiometric and UV/Vis spectrophotometric titrations. CV (Cyclic Voltammetry), DP-ASV (Differential Pulse-Anodic Stripping Voltammetry), ESI-MS experiments and quantum mechanical calculations have been also performed to derive information about the nature and possible structure of species formed. These results are also compared with those reported for the *8-HQA*/Fe^3+^ system in terms of chemical speciation and sequestering ability of *8-HQA*.

## 1. Introduction

**Scheme 1:**
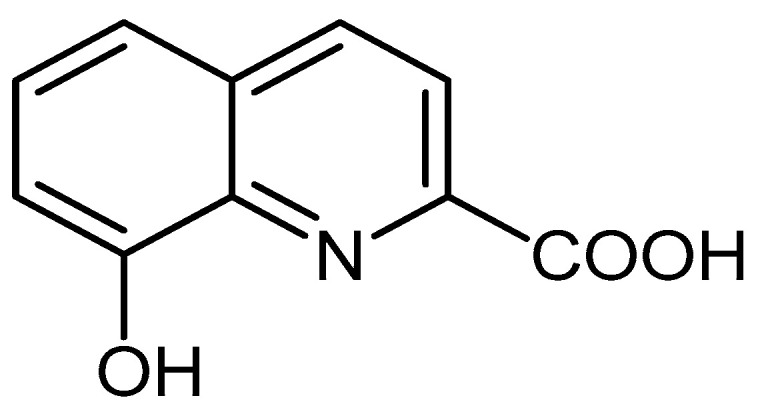
8-Hydroxyquinoline-2-carboxylic acid (8-HQA).

8-Hydroxyquinoline-2-carboxylic acid (*8-HQA*, also known as 8-hydroxyquinaldic acid, Scheme 1) was recently identified in the gut of Noctuid larvae (and in a few other lepidopterans) in high concentrations (0.5–5.0 mmol·dm^−3^) [1]. Being the end-metabolite of tryptophan, its biosynthesis seems to have an important role in the regulation of the microbiota of the referred larvae, most probably due to its action as a siderophore, as it shows very interesting properties as an iron chelator [2]. Despite the lack of knowledge on the natural occurrence of *8-HQA* in human biological fluids, recent findings reveal that it may have an impact on some physiological processes in the human body, especially in the colon, and in higher concentrations may also affect carcinogenesis and cancer progression [3].

A lot of work has been done on the use of 8-hydroxiquinoline (8-HQ) derivatives for several medicinal applications. In 1993, ABBOTT Laboratories developed a series of bifunctional chelators based on *8-HQA*, where the carboxylic acid is converted to an N-hydroxy-succinimidyl ester, which reacts with the side chain amino groups of proteins to link the bidentate chelator to the intended substrate for use as chelating agents in radiopharmaceutical applications, exploring different sizes, shapes and denticities according to the specific nature of the metal ion to be bound [4]. Furthermore, there are several 8-HQ derivatives reported as antimicrobial and antiparasitic, antioxidant, anti-neurodegenerative and anticancer agents. Their action occurs mainly through their chelating properties towards specific metal ions fundamental for triggering the mentioned diseases (e.g., Fe, Cu, Zn) [5,6,7]. Interestingly, *8-HQA* was recently identified by Capodagli et al. [8], using an enzymatic and structure-based assisted screening, as a selective, druglike, low-micromolar, non-competitive inhibitor of II FBA, a fructose 1,6-bisphosphate aldolase, essential within both Gram-positive and Gram-negative bacteria, as *Mycobacterium tuberculosis*, *Escherichia coli*, *Streptococcus pneumoniae* and *Candida albicans* among others. In vitro, 8-HQA has been identified as a selective II FBA inhibitor, with no inhibitory properties toward human and rabbit class I FBAs, presenting very promising properties as an anti-tuberculosis drug. Furthermore, in this particular case, as II FBA is a zinc metalloenzyme, the interaction between *8-HQA* and zinc seems to be a key point, together with the particular structural skeleton of the molecule [8].

As such, the study of the chemical speciation of *8-HQA* in aqueous solution (including natural waters and biological fluids) and its interactions with relevant metal ions seems to be a crucial aspect to get further insights about its action, not only as a potential drug but also on its role in the environment and living organisms.

Following our recent study on the sequestration of Fe^2+^ and Fe^3+^ by *8-HQA* [2], the present work reports the results of an investigation on the chemical speciation, coordination and sequestering ability of this ligand towards the molybdate ion (MoO_4_^2−^), in order to evaluate the possible role of *8-HQA* as molybdophore, other than siderophore. This assumption is supported by the fact that it is now clear that most siderophores can simultaneously act as other metallophores, as they are produced to overcome nutrient-limited conditions and to promote the uptake of other paramount elements than iron [9]. The choice of molybdate was taken because it is the main form of molybdenum in aqueous environments [10,11]. Molybdenum, as well as iron, has a vital functionality, being considered as an essential trace element for all higher plants, as well as animals and humans. In humans, molybdenum is found as a cofactor for, at least, four fundamental enzymes: sulfite oxidase, that catalyzes the terminal reaction in the degradation of sulfur amino acids cysteine and methionine; xanthine oxidase, catalyst of the oxidation of purines to uric acid; aldehyde oxidase, involved in the metabolism of various endo- and exogenous N-heterocyclic compounds; and mitochondrial amidoxime reducing component (mARC) that was just recently discovered, and seems to form the catalytic portion of a 3-component enzyme system with heme/cytochrome b5. Interestingly, the structure of mARC along with its high abundance in liver and kidney suggests that mARC could play a role in detoxification of N-hydroxylated substrates. Molybdenum requirements in humans are usually supplied by vegetables, especially legumes, grains, and nuts; deficiency is responsible for several human diseases, including neurological abnormalities, lens dislocation of the eyes, major dysmorphic features of the head and, finally, it appears to be correlated with amyotrophic lateral sclerosis [10,11,12,13,14,15,16,17,18,19].

In order to derive a comprehensive set of information necessary to assess the chemical speciation of the *8-HQA*/MoO_4_^2−^ system, as well as the coordination behavior and the sequestering ability of *8-HQA* towards molybdate, a multi-technique approach needs to be adopted. ISE-H^+^ (glass electrode) potentiometric and UV/Vis spectrophotometric titrations were performed, in KCl_(aq)_ at *I* = 0.2 mol·dm^−3^ and *T* = 298.15 K. Voltammetric and MS experiments, as well as quantum mechanical studies, were also performed. Combining the results of all the above techniques it was possible to define the *8-HQA*/MoO_4_^2−^ system in terms of number, type and mode of coordination of the species formed. A general assessment of the sequestering ability of *8-HQA* towards molybdate under different pH conditions and in comparison with the *8-HQA*/Fe^3+^ system is also presented.

## 2. Materials and Methods

### 2.1. Chemicals

8-Hydroxy-2-quinolinecarboxylic acid (*8-HQA*, L^2−^) solutions were prepared by weighing the commercial product. A minimum known amount of EtOH was used to promote initial ligand solubilization in water. This percentage never exceeded 1.5–2.0% (*v*/*v*). MoO_4_^2−^ solutions were prepared by weighing the dipotassium salt. The purity of both *8-HQA* and MoO_4_^2−^ was checked potentiometrically by alkalimetric titrations, resulting always ≥99%. KCl aqueous solutions were prepared by weighing the pure salt, previously dried in an oven at *T* = 383.15 K for at least 2 h. HCl and KOH solutions were prepared by diluting the concentrated ampoules and were standardized against sodium carbonate and potassium hydrogen phthalate, respectively, previously dried in an oven at *T* = 383.15 K for at least 2 h. KOH solutions were stored in dark bottles and preserved from atmospheric CO_2_ by means of soda lime traps. All the solutions were prepared with analytical grade water (R = 18 MΩ cm^−1^) using grade A glassware and were used immediately after their preparation. All chemicals were purchased from Sigma-Aldrich, Italy, at their highest available purity. LC-MS grade acetonitrile was purchased from Merck Life Science (Merck KGaA, Darmstadt, Germany).

### 2.2. Apparatus and Procedure for Potentiometric Measurements

ISE-H^+^ potentiometric titrations have been performed by two operators, using two different apparatus and totally independent reagents, in order to minimize systematic errors. The first apparatus was a Metrohm model 809 Titrando system, equipped with a half-cell glass electrode (Ross type 8101, from Thermo-Orion) and a double-junction reference electrode (type 900200, from Thermo-Orion). The second apparatus consisted of a Mettler Toledo DL50 titrator, equipped with a Schott Instruments N6180 combination glass electrode. The Metrohm TiAMO 2.5 and LabX 1.3 software were used for the first and second apparatus, respectively, to control and record all the parameters of an automatic titration and, in particular, titrant delivery, data acquisition, and e.m.f. stability. Potentiometric titrations were performed at *T* = 298.15 ± 0.1 K in thermostatted cells under magnetic stirring and bubbling purified pre-saturated N_2(g)_ through the solution, in order to exclude O_2(g)_ and CO_2(g)_ inside. The titrand solution consisted of different amounts of *8-HQA* (0.4 ≤ *c_8-HQA_*_/_mmol·dm^−3^ ≤ 1.5), molybdate (0.4 ≤ *c_MoO4_*/mmol·dm^−3^ ≤ 1.5), a slight excess of hydrochloric acid (1.0 ≤ *c*_H_/mmol·dm^−3^ ≤ 10.0), and KCl in order to obtain the pre-established ionic strength value (*I* = 0.2 mol·dm^−3^). Measurements were performed considering different concentration ratios, i.e., 1:1 ≤ *c*_MoO4_:*c_8-HQA_* ≤ 1:3. 25 or 50 cm^3^ of titrand solution were titrated by standard KOH to pH ≈ 10.5–11. For each experiment, electrode calibrations were performed by independent titrations of HCl with standard KOH under the same ionic strength conditions as the systems to be investigated, in order to determine the electrode potential (*E*^0^) and the acidic junction potential (*E*_j_ = *j*_a_[H^+^]). By this procedure, the pH scale used is the free scale, pH ≡ −log [H^+^], where [H^+^] is the free proton concentration (not activity). The reliability of electrode calibration in the alkaline pH range was checked by determining appropriate p*K*_w_ values. A total of 80–100 data points were collected for each titration, and the equilibrium state during titrations was checked by adopting the usual precautions, such as the check of the time required to reach equilibrium and the execution of back titrations.

### 2.3. Apparatus and Procedure for Spectrophotometric Measurements

UV/Vis spectrophotometric titrations were carried out using a Varian Cary 50 UV/Vis spectrophotometer equipped with an optical fiber probe with a fixed 1 cm path length. The spectrophotometer was connected to a PC and the data acquisition of the couples absorbance (*A*) vs. wavelength (*λ*/nm) was made using Varian Cary WinUV (version 3.00) software. Measurements were performed in thermostatted cells at *T* = 298.15 ± 0.1 K in the wavelength range 200 < *λ*/nm ≤ 800. During the measurements, a 602 Metrohm Biotrode combined metrosensor glass electrode was also introduced in the cell, and was connected to a Model 713 Metrohm potentiometer. In this way, it was possible to simultaneously record both the *A* vs. *λ* and e.m.f. vs. titrant volumes for each alkalimetric titration point. The combined glass electrode was calibrated before each experiment in terms of free proton concentration, as reported in previous section. The titrant was delivered in the measurement vessel by means of a 665 Metrohm motorized burette. Solutions were maintained under magnetic stirring and N_2(g)_ was bubbled in order to exclude the presence of CO_2(g)_ and O_2(g)_, paying attention to avoid interference with both the electrode and the optical fiber probe. Spectrophotometric measurements were carried out by titrating 25 cm^3^ of the titrand solution with standard NaOH solutions, up to pH ≈ 10.5–11. The titrand solutions consisted of different amounts of *8-HQA* and molybdate (10^−6^ ≤ *c*_x*/*_mmol·dm^−3^ ≤ 10^−5^, only one of them for the determination of their protonation constants), hydrochloric acid (1.0 ≤ *c*_H_/mmol·dm^−3^ ≤ 10.0), and KCl in order to obtain the pre-established ionic strength value (*I* = 0.2 mol·dm^−3^). As for potentiometric measurements, titrand solutions were prepared considering different concentration ratios, i.e., 1:1 ≤ *c*_MoO4_:*c_8-HQA_* ≤ 1:3.

### 2.4. Apparatus and Procedure for Voltammetric Measurements

Cyclic Voltammetry (CV) and Differential Pulse-Anodic Stripping Voltammetry (DP-ASV) experiments were carried out (at *T* = 298.15 ± 0.1 K in thermostatted cells, at *I* = 0.2 mol·dm^−3^ in KCl_(aq)_) by a Metrohm 663 VA Stand (Series 05) workstation, equipped with a three electrode system supplied by Metrohm, consisting of: a Multimode Mercury Electrode (MME, code 6.1246.020) working in SMDE mode (Static Mercury Drop Electrode), a double junction Ag/AgCl/KCl (3 mol·dm^−3^) reference electrode (RE) (model 6.0728.000 + 6.1245.000), and a glassy carbon (GC) auxiliary electrode (AE) (model 6.1247.000). The MME was filled with 99.9999% Mercury (electronic grade, from Sigma-Aldrich). The workstation was connected to an Autolab potentiostat/galvanostat (Eco Chemie) with an IME663 interface (Eco Chemie). The whole system was controlled using NOVA v. 1.10 software (Metrohm). The free hydrogen ion concentration in the DP-ASV and CV experiments was measured before and after each voltammetric run by using the same apparatus and procedure already described in the previous sections. Purified N_2(g)_ was bubbled into the solutions for 300 s prior to any experiment.

CV measurements were performed on 25 cm^3^ of solutions containing known amounts of molybdate (10^−4^ ≤ *c*_MoO4_ ≤ 5 × 10^−4^ mol·dm^−3^), *8-HQA* in suitable concentrations to reach desired *8-HQA*:MoO_4_^2−^ concentration ratios (1:1 ≤ *c*_MoO4_:*c_8-HQA_* ≤ 1:3), KCl in order to obtain the pre-established ionic strength value (*I* = 0.2 mol·dm^−3^), and adding KOH or HCl to adjust pH at the desired values (3.5 < pH < 10.5). Two series of CV measurements were performed for various *8-HQA*:MoO_4_^2−^ ratios: in one, different scan rates (20–500 mV/s) were applied to solutions at fixed pH (pH = 7.3); in the other, scan rate was fixed at 100 mV/s and pH was varied (3.5 < pH < 10.5). Scan conditions for the first CV series were: starting potential = ending potential = 0.000 V, upper vertex potential = 0.050 V, lower vertex potential = −0.400 V, number of stop crossings 10, step potential = −0.008 V. For the second CV series they were: starting potential = ending potential = −0.020 V, upper vertex potential = 0.000 V, lower vertex potential = −1.000 V, number of stop crossings 10, step potential = −0.008 V.

DP-ASV measurements were performed on 25 cm^3^ of solutions containing known amounts of molybdate (10^−4^ ≤ c_MoO4_ ≤ 5 × 10^−4^ mol·dm^−3^), KCl in order to obtain the pre-established ionic strength value (*I* = 0.2 mol·dm^−3^), KOH or HCl to adjust pH at the desired values (3.5 < pH < 10.5), and adding *8-HQA* into the solutions up to *8-HQA*:MoO_4_^2−^ ~3:1. Voltammograms were recorded after each *8-HQA* addition, while pH was checked both before and after *8-HQA* additions. Working conditions were as follows: deposition potential = −1.000 V, deposition time = 2 s, equilibration time 10 s, start potential = −1.000 V, end potential = 0.000 V, step potential = 0.005 V, modulation amplitude = 0.05 V, interval time = 0.5 s, scan rate 10 mV/s. Further experimental details on voltammetric measurements can be found in refs. [20,21,22].

### 2.5. Apparatus and Procedure for ESI-MS Measurements

ESI-MS analyses were performed on a LCMS-8050 triple quadrupole mass spectrometer, through an ESI source, coupled to a Nexera-e liquid chromatograph (Shimadzu, Kyoto, Japan), consisting of a CBM-20A controller, a LC-30AD dual-plunger parallel-flow pump, a DGU-20A_5_R degasser, a CTO-20AC column oven, and a SIL-30AC autosampler. Instead of a column, a 0.13 mm I.D. capillary tube was used to connect the autosampler to the mass spectrometer. Injection volume was 1 μL and flow rate was 0.5 mL min^−1^ of pure acetonitrile. Scan range was 60–900 *m*/*z*, with a scan speed of 10,000 u s^−1^. Event time was 0.100 s. ESI source was used in both negative and positive ionization modes. Nebulizing gas flow = 3 L min^−1^, heating gas flow = 5 L min^−1^, interface temperature = 573 K, DL temperature = 523 K, heat block temperature = 673 K, drying gas flow = 5 L min^−1^, interface voltage = 3 kV.

For selected conditions, some High Resolution Mass Spectra (HRMS) measurements were also performed, as reported elsewhere [2], to give further confirmation about peaks.

Analyzed aqueous solutions consisted of different amounts of potassium molybdate (5 × 10^−4^ ≤ *c*_MoO4_ ≤ 10^−3^ mol·dm^−3^), *8-HQA* in suitable concentrations to reach desired *8-HQA*:MoO_4_^2−^ concentration ratios (1:1 ≤ *c*_MoO4_:*c_8-HQA_* ≤ 1:3), and HCl or NaOH to adjust pH (2.5 < pH < 11, measured by the same apparatus above described). Samples were freshly prepared and injected immediately after preparation.

### 2.6. Procedure for Quantum Mechanical Calculations

Quantum mechanical computations in this study were carried out at the density functional theory (DFT) level with Gaussian09 (Revision C01) [23]. Geometry optimizations of the complexes were performed using the B3LYP hybrid functional, using the effective core potential LanL2DZ and 6-31+G(d) basis sets for the metal and nonmetal atoms, respectively. Solvation (water) effects were accounted through both the Integral Equation Formalism Polarizable Continuum Model (IEFPCM) [24] and the Conductor-Like Polarizable Continuum Model (CPCM) [25,26]. Furthermore, within the PCM formalism, both the UFF and the Bondi cavity radii have been tested, as well as different reaction field correction factors (alpha = 1.1 and 1.2), though negligible differences have been obtained. Based on experimental findings and previous calculations, the metal atom was always considered in its +6 oxidation state, Mo(VI), corresponding to singlet spin multiplicity.

### 2.7. Thermodynamic Calculations

All the parameters of the potentiometric titrations (analytical concentrations of reagents, *K*_w_, *E^0^*, liquid junction potential coefficient *j_a_*) were refined by using the non-linear least squares computer program BSTAC [27]. BSTAC was also used to refine the complex formation constants, in parallel with Hyperquad2013 program of the Hyperquad suite [28]. UV/Vis spectra were analyzed by the HypSpec2014 program of the same suite, which allowed the determination of the stability constants and the molar absorbance spectra of each absorbing species. The ES4ECI [27] and HYSS [28] programs were used to draw the speciation and sequestration diagrams and to calculate the species formation percentages. The sequestering ability of *8-HQA* towards molybdate (and Fe^3+^ for comparison) has been quantified by means of pL_0.5_ calculations in various conditions. As detailed elsewhere [29], pL_0.5_ is a semiempirical parameter representing the total concentration of a ligand (*L*, as −log *c_L_*) necessary to bind 50% (as mole fraction, *x_M_* = 0.5) of a given component (*M*) in a given solution when *c_M_* γ 0. By plotting the fraction of *M* (fixing *c_M_* = 10^−24^) bound to *L* vs. −log *c_L_* a sigmoidal curve is obtained (sequestration diagram), and can be fitted to the Boltzmann-type equation:

(1) $$x_{M}=\frac{1}{{1+10}^{{(\mathrm{pL}-\mathrm{pL}}_{0.5})}}$$

where pL = −log *c*_L_, and pL_0.5_ is the only adjustable parameter. Like other “p” functions (e.g., the pM), the higher the pL_0.5_, the greater the sequestering ability.

Within the manuscript, if not differently specified, protonation constants of molybdate (q = 0) and *8-HQA* (p = 0) and complex formation constants are given according to the overall equilibrium:

p MoO_4_^2−^ + q *8-HQA*^2−^ + r H^+^ = (MoO_4_)_p_(*8-HQA*)_q_H_r_^(2p+2q−r)−^       log *β*_pqr_ (2)

Concentrations, ionic strengths, protonation and complex formation constants are expressed in the molar (*c*, mol·dm^−3^) concentration scale. Uncertainties are reported at the ±95% confidence interval.

## 3. Results and Discussion

### 3.1. Determination of the Speciation Model. Nature and Stability of (MoO_4_)_p_(8-HQA)_q_H_r_^(2p+2q−r)−^ Complexes: Potentiometric and Spectrophotometric Investigation

#### 3.1.1. Acid–Base Properties of MoO_4_^2−^ and 8-HQA

For an accurate determination of the stability constants of (MoO_4_)_p_(*8-HQA*)_q_H_r_^(2p+2q−r)−^ complexes, the acid–base properties of both MoO_4_^2−^ and *8-HQA* should be known in the exact conditions of the experiments. Protonation constants at *T* = 298.15 K and *I* = 0.2 mol·dm^−3^ in KCl_(aq)_ were taken from previous works for both ligands [2,30], and are reported as supplementary information (Table S1). Furthermore, in order to analyze data from spectrophotometric measurements, the specific molar absorbances of both the free ligands and their protonated species must be also known, to be added as input in HypSpec2014 software. To this aim, dedicated experiments have been performed in this work for both ligands. The analysis of data from UV/Vis spectrophotometric titrations gave, within the experimental error, protonation constants that are in excellent agreement with previous findings, as shown comparing values reported in Table 1 with those of Table S1.

As already observed, polymeric molybdate species are not formed under the conditions of UV/Vis experiments, since they become significant only at higher concentrations [30]. For both the free ligands and their protonated species, specific molar absorbances determined in these experiments are reported in Table S2 and plotted in Figure S1 in the wavelength range 205 ≤ *λ*/nm ≤ 495 (although spectra were recorded in the range 200 ≤ *λ*/nm ≤ 800, unnecessary wavelengths have been cut during calculations to reduce noise and useless information).

#### 3.1.2. Stability Constants of (MoO_4_)_p_(8-HQA)_q_H_r_^(2p+2q−r)−^ Species

The analysis of potentiometric and spectrophotometric data evidenced the formation of differently protonated 1:1 species, namely (MoO_4_)(*8-HQA*)^4−^, (MoO_4_)(*8-HQA*)H^3−^, (MoO_4_)(*8-HQA*)H_2_^2−^ and (MoO_4_)(*8-HQA*)H_3_^−^. The corresponding stability constants are reported in Table 2.

**Figure 1:**
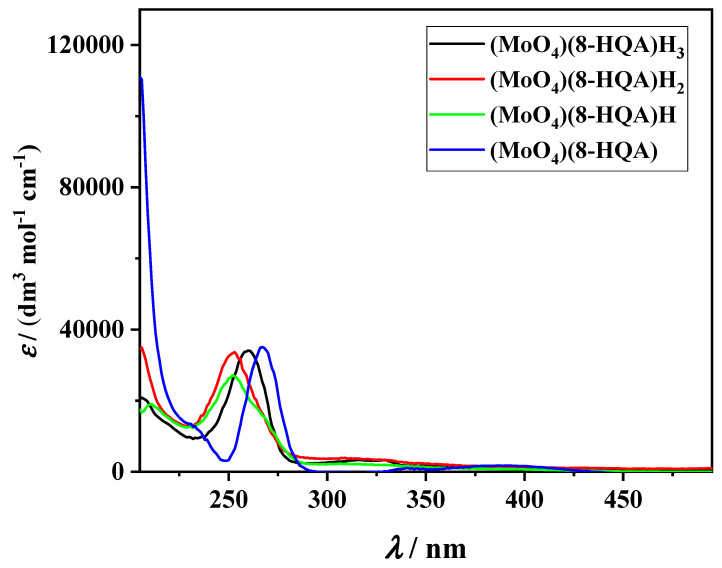
Specific molar absorbances of (MoO_4_)_p_(*8-HQA*)_q_H_r_ species at different wavelengths, at *T* = 298.15 K.

Spectrophotometric data analysis (an example of UV/Vis titration is reported in Figure S2) allowed further calculation of the specific molar absorbances of (MoO_4_)_p_(*8-HQA*)_q_H_r_ species, plotted in Figure 1 (and reported in Table S3) in the wavelength range 205 ≤ *λ*/nm ≤ 495.

**Figure 2:**
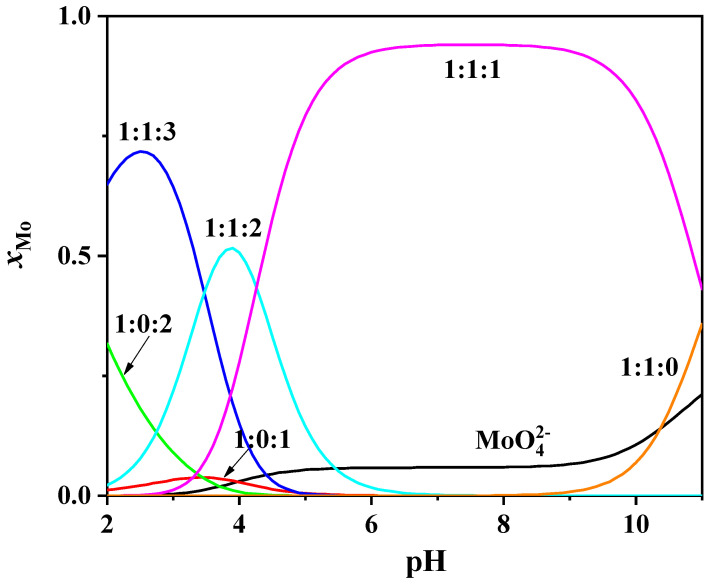
Distribution of molybdate species vs. pH, in the presence of *8-HQA*. p:q:r indexes refer to (MoO_4_)_p_(*8-HQA*)_q_H_r_^(2p+2q−r)−^. Conditions: *c*_MoO4_ = *c_8-HQA_* = 1 × 10^−5^ mol·dm^−3^, *T* = 298.15 K and *I* = 0.2 mol·dm^−3^ in KCl_(aq)_.

As observed from Table 2, the agreement between results obtained by both techniques is very good, especially in relation to the fact that concentrations used for the two set of measurements differ from each other by some orders of magnitude, which could favor the formation of different species (e.g., species with p and/or r > 1). The same table reports a further column with proposed stability constants, which derive from the average of both sets, including uncertainties. The use of those averaged constants results, in the worst case (i.e., for the (MoO_4_)(*8-HQA*)H_2_^2−^ at its maximum formation, at pH ~3.9, see below), in an uncertainty of about 5% in terms of formation percentages of the species (with respect to values coming from single datasets). Those constants were used to draw the speciation diagram of molybdate in the presence of *8-HQA*, shown in Figure 2.

The distribution of the species does not change significantly at higher concentrations (e.g., *c*_MoO4_ = *c_8-HQA_* = 1 × 10^−3^ mol·dm^−3^, Figure S3) or at different MoO_4_^2−^:*8-HQA* ratios (e.g., *c*_MoO4_:*c_8-HQA_* = 1:2, Figure S4). Uncomplexed molybdate species are still present over almost the whole pH range, which however becomes insignificant at higher concentrations (~1–2%, see Figure S3). In any case, in the presence of *8-HQA*, molybdate is almost entirely complexed by this ligand, with uncomplexed species never exceeding a percentage of ~5%, except in the very acidic and very basic investigated pH ranges, where the MoO_4_H_2_ and the free MoO_4_^2−^ reach ~30% and ~20% at pH = 2.0 and 11.0, respectively. This is already a good indication that *8-HQA* could be a good sequestering agent towards molybdate, an aspect that is discussed later in the dedicated section. Concerning the importance of single species, the monoprotonated (MoO_4_)(*8-HQA*)H^3−^ is so far the most important, dominating molybdate speciation in the pH range 4 < pH < 10, while di- and tri-protonated (MoO_4_)(*8-HQA*)H_2_^2−^ and (MoO_4_)(*8-HQA*)H_3_^−^ species reach their maximum at pH ~3.9 and pH < 2.5, respectively. The fully deprotonated (MoO_4_)(*8-HQA*)^4−^ species becomes significant at pH > 10.0–10.5.

One of the first considerations that spontaneously comes out from analyzing the obtained speciation scheme is that only monomeric (MoO_4_)(*8-HQA*)H_r_ species were determined. This result appeared a bit surprising if one takes into account results previously obtained for Fe^2+^/*8-HQA* and Fe^3+^/*8-HQA* systems (which form very stable Fe(*8-HQA*)_2_ and, only for Fe^3+^, Fe(*8-HQA*)_3_ species [2]), as well as the fact that both potentiometric and spectrophotometric titrations were designed in such a way to promote the formation of (MoO_4_)(*8-HQA*)_q_H_r_ species with q > 1 (measurements with up to *c*_MoO4_:*c_8-HQA_* = 1:3 ratios were considered). This at first glance surprising observation gave rise to a series of questions related to the speciation scheme proposed and, eventually, on the coordination of molybdate and the binding modes of *8-HQA*.

This led to the planning of new investigations by different techniques to try to get further insights about those aspects.

### 3.2. Rebuttal of the Speciation Model. Towards Poly-8-HQA Complexes: Voltammetric Investigation

In addition to the classical information on the electrochemical behavior of investigated compounds, voltammetric techniques prove also to be, for their characteristics, very useful to derive information on the nature and stability of complexes formed by an electrochemically active compound M (usually a metal cation, but, in general, any component undergoing redox reactions at the working electrode) with a ligand L. In particular, since the pioneering studies of Lingane [31] and De Ford and Hume [32], it is known that the deposition potential of an electroactive component undergoes negative shifts in the presence of complexing ligands, and that these shifts depend on the nature and stability of the formed complex(es). If properly designed, voltammetric measurements can be therefore exploited to perform chemical speciation studies and to determine the stability constants of ML_q_H_r_ species, especially when q > 1 [21,33].

**Figure 3:**
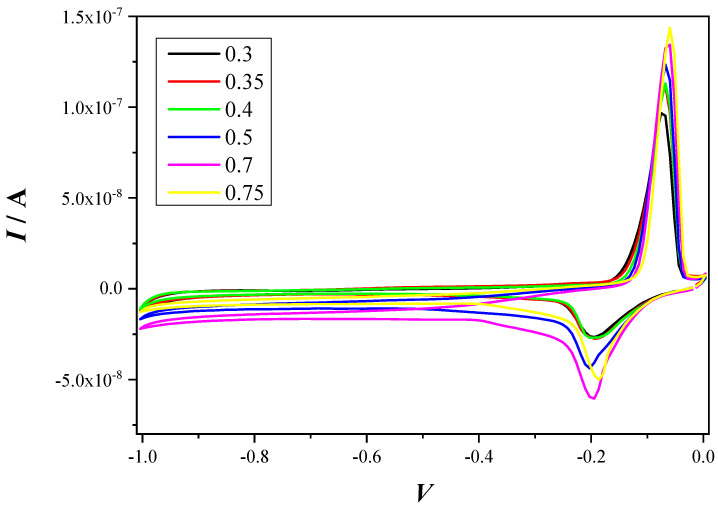
CVs (Cyclic Voltammetry) of molybdate in the presence of *8-HQA* at different ratios. Conditions: *c*_MoO4_ = 2 × 10^−4^ mol·dm^−3^, *T* = 298.15 K and *I* = 0.2 mol·dm^−3^ in KCl_(aq)_, pH = 7.3, scan rate 100 mV/s. Captions in figure refer to *8-HQA* concentration, in mmol·dm^−3^.

In this work, preliminary scans were performed to verify the absence, for *8-HQA,* of redox reactions in the investigated potential range. Then, a series of cyclic voltammograms were recorded in various conditions of pH, molybdate concentration, *c*_MoO4_:*c_8-HQA_* ratios and at different scan speeds, as reported in the experimental section (some CVs are shown in Figures S5, S6 and Figure 3).

As can be noted, in the experimental conditions adopted, the electron(s) transfer processes at electrode (HDME) surface is quasireversible, which makes data analysis slightly more complex and does not allow an immediate definition of molybdate reduction process in these conditions. Furthermore, even the mere electrochemical behavior of molybdenum in aqueous solution is not straightforward, due to the high number of possible oxidation states (from +6 to +2) of this element in aqueous solution, which are also conditions/speciation dependent (i.e., pH, Mo concentration) [34].

**Figure 4:**
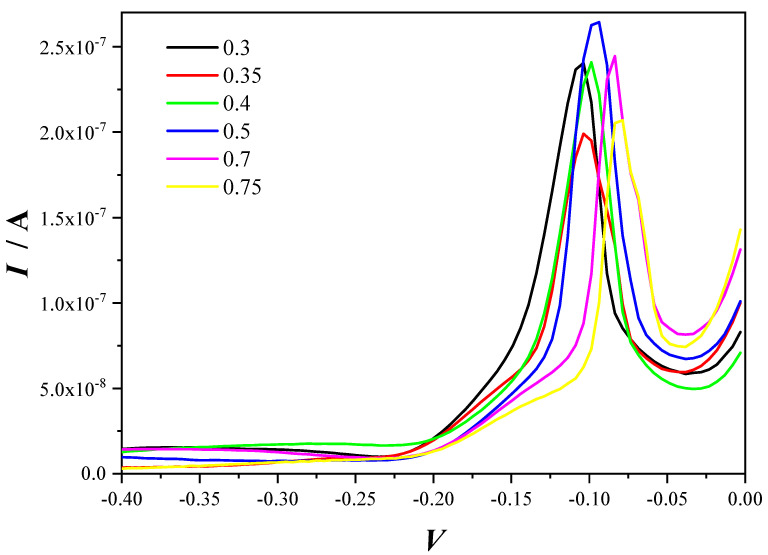
DP-ASVs (Differential Pulse-Anodic Stripping Voltammetry) of molybdate in the presence of *8-HQA* at different ratios. Conditions: *c*_MoO4_ = 2 × 10^−4^ mol·dm^−3^, *T* = 298.15 K and *I* = 0.2 mol·dm^−3^ in KCl_(aq)_, pH = 7.3, scan rate 10 mV/s. Captions in figure refer to *8-HQA* concentration, in mmol·dm^−3^.

Independently of this, the quasireversibility of the process made possible the use of DP-ASV for the initial purpose of this investigation, i.e., the verification of the presence of more than one (MoO_4_)(*8-HQA*)_q_H_r_ species and, possibly, of poly-*8-HQA* complexes. Figure 4 reports a series of superimposed voltammograms obtained in the same conditions of CVs shown in Figure 3 (i.e., at pH = 7.3, fixed molybdate concentrations and different *c*_MoO4_:*c_8-HQA_* ratios).

**Figure 5:**
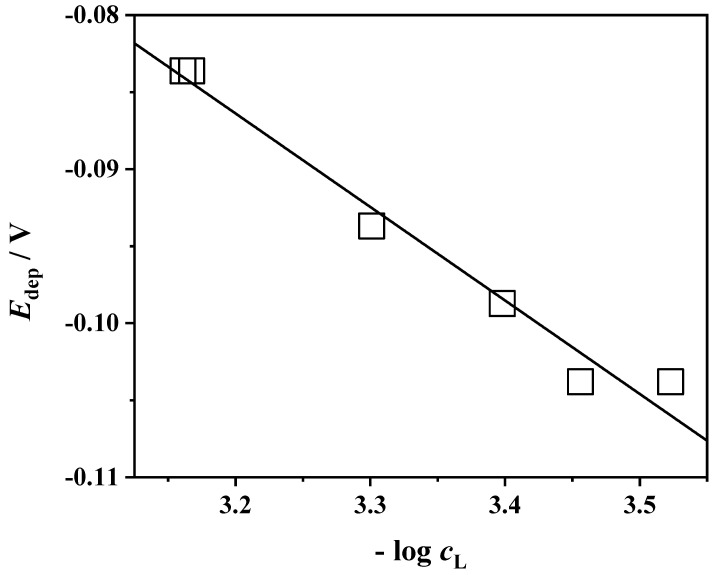
Deposition potential of molybdate vs. *8-HQA* concentration (in mol·dm^−3^, as -log *c_8-HQA_*) in DP-ASV measurements. Conditions: *c*_MoO4_ = 2 × 10^−4^ mol·dm^−3^, *T* = 298.15 K and *I* = 0.2 mol·dm^−3^ in KCl_(aq)_, pH = 7.3, scan rate 10 mV/s.

As known, complexation (by *8-HQA*) affects in these cases both the deposition potential and the current peak. The number of complexes formed can be obtained by plotting the deposition potential as a function of the concentration of *8-HQA* in solution, as shown in Figure 5.

The straight line obtained (*R^2^* = 0.98) is a clear indication of the formation of only one complex [33]. Then, for reversible systems and consecutive ML_q_ complexes, the slope of such a line (that, in this case, it is −0.060 V) would have been dependent on the q/*n* ratio, being q the number of ligands bound to the electroactive species and *n* the number of electrons exchanged in the process [6]. However, this is not valid for quasireversible processes, so that the only sure information that is possible to obtain is, again, only the number of complexes formed (i.e., only one), but not the stoichiometry.

### 3.3. Identification of (MoO_4_)_p_(8-HQA)_q_H_r_ Species and Coordination of Mo(VI): ESI-MS Investigation

Mass spectrometric techniques are becoming very frequent in chemical speciation studies to identify possible species. However, it is important to point out here that they cannot provide, alone, unquestionable results, as it happens for all other techniques in which the chemical and physical conditions of original solutions are somehow altered before and/or during experiments (a classical debated example is given by crystallography, in which the evaporation of the solvent leads to a progressive concentration of components until the formation of the crystal, which does not always reflect the situation of the diluted systems). Nevertheless, equally unquestionable is the fact that mass spectrometry can offer very useful information that, in combination with other results, can support various hypotheses.

In the present work, a series of ESI-MS spectra were recorded for molybdate/*8-HQA* solutions at different pH and *c*_MoO4_:*c_8-HQA_* ratios, as described in the experimental section. Some examples are reported in Figures S7–S11. Even before entering into the details of the analysis of single peaks, what is immediately evident is that MS spectra at different ratios are perfectly superimposable (apart from relative abundancies, Figures S7–S10), which reflects the great similarities among the analyzed solutions and, therefore, their chemical speciation. Similar considerations can be made when analyzing solutions at the same *c*_MoO4_:*c_8-HQA_* ratio, but at different pH (Figure S11). Apart from the relative abundancies of peaks, the spectra at various pH are almost identical in structure. This last aspect should not be surprising, since it is well known that ESI sources involve protons during the ionization process, so that it is hard to distinguish species which are identical in structure but only different for the number of bound protons. However, this further result, apparently “negative”, brings out another interesting consideration: it is very probable that (MoO_4_)_p_(*8-HQA*)_q_H_r_ species initially present in the injected solutions at various pH have the same “structure”, with bound protons affecting the coordination only marginally. If this is plausible, it gives rise to other questions on the coordination of molybdenum.

It is reported that, during complexation, Mo(VI) in molybdate often expands its coordination, usually from 4 (tetrahedral) to 6 (octahedral) [35]. Moreover, several structures have been reported in which strong binding, e.g., by catecholates or hydroxopyridinones, leads to MoO_3_ and/or MoO_2_^2+^ moieties [36,37,38]: every oxygen atom from the original molybdate is “lost” during complexation through the formation of a water molecule that, from a mere stoichiometric point of view, corresponds to the involvement in the formation equilibrium of two protons per “lost” oxygen (of course they can be donated by the ligand itself).

MoO_4_^2−^ + 2 H^+^ + *L* = (MoO_3_)*L* + H_2_O    ≡ (MoO_4_)*L*H_2_ (3)

MoO_4_^2−^ + 4 H^+^ + *L* = (MoO_2_)*L* + 2 H_2_O  ≡ (MoO_4_)*L*H_4_ (4)

Therefore, in this regard, there is the possibility that, of the four (MoO_4_)_p_(*8-HQA*)_q_H_r_ species determined, the (MoO_4_)(*8-HQA*)H_2_ (and eventually (MoO_4_)(*8-HQA*)H_3_) could correspond to the (MoO_3_)(*8-HQA*) (and (MoO_3_)(*8-HQA*)H), while this should not be stoichiometrically possible for the monoprotonated (MoO_4_)(*8-HQA*)H and the fully deprotonated (MoO_4_)(*8-HQA*) species. This further issue can be solved by a more careful analysis of recorded mass spectra, through the identification of single signals, with the additional support of some extra HRMS measurements to identify the isotopic patterns.

**Figure 6:**
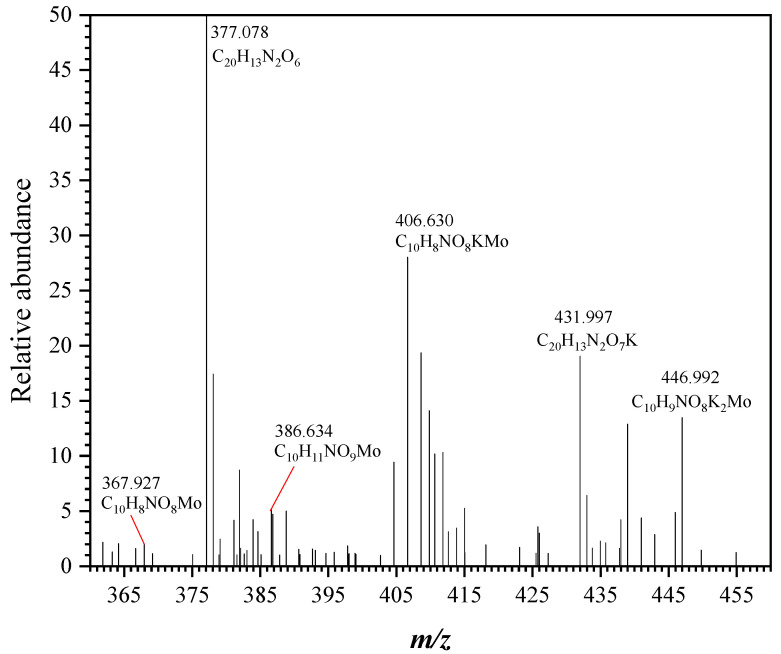
HRMS (High Resolution Mass Spectra) spectrum of molybdate/*8-HQA* solutions at *c*_MoO4_:*c*_8-HQA_ = 1:2 ratio.

Figure 6 reports an example of HRMS spectrum obtained from a solution at *c*_MoO4_:*c*_8-HQA_ = 1:2. In this figure, only the region with 360 ≤ *m/z* ≤ 460 is shown because that is of interest for complex species. Spectra at *m/z* < 360 are characterized by “uncomplexed” and differently protonated molybdate and *8-HQA* adducts, including ion pairs with K^+^, such as: *m/z* = 144.045 ([C_9_H_6_NO], corresponding to the monoprotonated *8-HQA* with lost carboxylate), *m/z* = 162.849 ([MoO_4_H]), *m/z* = 188.035 ([C_10_H_6_NO_3_], i.e., the monoprotonated *8-HQA*), *m/z* = 225.991 ([C_10_H_5_NO_3_K], i.e., the free *8-HQA* with K^+^), *m/z* = 228.006 ([C_10_H_7_NO_3_K], i.e., the diprotonated *8-HQA* with K^+^), *m/z* = 242.284 ([MoO_4_H_3_K_2_]), *m/z* = 265.961 ([C_10_H_6_NO_3_K_2_], i.e., the monoprotonated *8-HQA* with 2 K^+^). Even at *m/z* > 350 it is still possible to detect “free” *8-HQA* adducts, like the signal at *m/z* = 377.078, corresponding to [C_20_H_13_N_2_O_6_] (i.e., the tri-protonated dimer of *8-HQA*), and *m/z* = 431.997 (i.e., [C_20_H_13_N_2_O_7_K], the monohydrated, monoprotonated dimer with K^+^). The presence of these signals is worth noting because, despite these ligand dimeric species being observed with high intensities, any signal was detected in all the spectra for eventual dimers containing molybdate in all the investigated *m/z* range. According to Figure 6, in the *m/z* range of interest, the following signals were identified, corresponding to molecular adducts containing one at least one molecule of molybdate, one of *8-HQA* and one of water. In particular, isotopic patterns of peaks at *m/z* = 367.927 correspond to [C_10_H_8_NO_8_Mo], i.e., (MoO_4_)(*8-HQA*)H(H_2_O); *m/z* = 386.634 is [C_10_H_11_NO_9_Mo], i.e., (MoO_4_)(*8-HQA*)H_2_(H_2_O)_2_; *m/z* = 406.630 is [C_10_H_8_NO_8_KMo], i.e., (MoO_4_)(*8-HQA*)H(H_2_O)K; *m/z* = 446.992 is [C_10_H_9_NO_8_K_2_Mo], i.e., (MoO_4_)(*8-HQA*)H_2_(H_2_O)_2_K_2_. Other than the fact that these findings are in perfect agreement with the speciation scheme proposed, the most important aspect to consider is that no adducts compatible with the presence of MoO_3_ and/or MoO_2_^2+^ moieties were observed in any of the spectra recorded under all the investigated conditions, as well as for eventual presence of poly-*8-HQA* molybdate complexes. Again, though this is not an absolute proof of the presence/absence of these species, this finding represents a further evidence in support of the correctness of the proposed speciation model.

### 3.4. Possible Binding Modes: Quantum Mechanical Calculations

In order to get further insights on the binding modes and the structure of the (MoO_4_)_p_(*8-HQA*)_q_H_r_ species, quantum mechanical calculations have been performed. Based on previous findings on the coordination of *8-HQA* on Fe [2], in which this ligand can act both as tri- and bi-dentate to form very stable complexes, various configurations have been tested. Several DFT calculations were performed starting from possible structures with one *8-HQA* bound to molybdate through three binding sites (i.e., one carboxylic oxygen “O_c_”, the nitrogen, and the hydroxyl oxygen “O_h_”), and through two of them (i.e., O_c_ and N, O_h_ and N and, though less probable, O_c_ and O_h_). However, upon structural optimization none of these hypothetical configurations led to a stable complex structure: either energy convergence was never reached or, in a few cases, the obtained minimized structure showed *8-HQA* bound to molybdate through one binding site only (usually through O_h_) and, therefore, a five-fold coordination of molybdenum. Moreover, some attempts were also performed including two *8-HQA* molecules in the initial complex configuration, or replacing MoO_4_ with either MoO_3_ or MoO_2_^2+^ moiety, with analogous unsatisfactory results.

**Figure 7:**
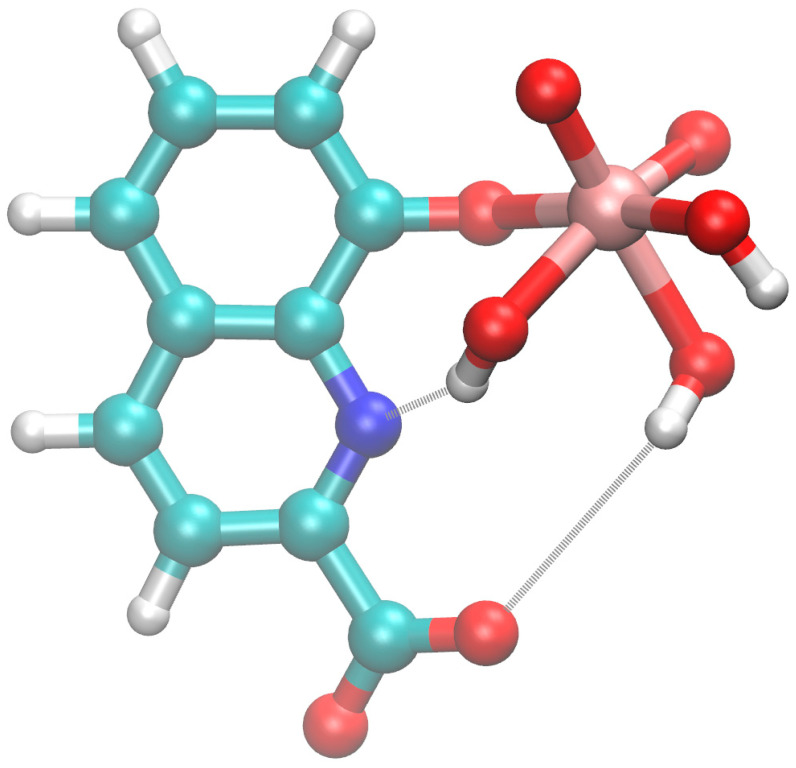
Optimized structure of the (MoO_4_)(*8-HQA*)H(H_2_O) species proposed from DFT calculations.

Then, based on ESI-MS results, some attempts were made considering the addition of one water molecule coordinated to molybdate (i.e., OH + H), thus leading to an interesting, stable complex structure for the (MoO_4_)(*8-HQA*)H(H_2_O) species, as depicted in Figure 7.

According to this configuration, two hydroxyl groups bound to Mo(VI) are close enough to form relatively strong hydrogen bonds with the nitrogen and carboxylic oxygen of *8-HQA*, thus supporting the high stability constant of the corresponding complex species. At the same time, such a configuration is compatible with the formation, upon pH change, of both the less protonated (MoO_4_)(*8-HQA*) species (through deprotonation of one OH bound to Mo) and the di- and tri- protonated ones (via protonation of *8-HQA* carboxylate and nitrogen). Besides, it is worth noting that the proposed structure (Figure 7) is also nicely consistent with other requirements or observations about the Mo complex, such as the formal net charge of the complex (i.e., z = −3, the extra water molecule giving no electric contribution), the hexavalent oxidation state of Mo and its six-fold coordination (i.e., the octahedral geometry is the most commonly observed in Mo(VI) complexes).

As a final consideration, the above structure highlights the important role of water in the stabilization and formation of the complex, an observation that may open new perspectives in the study and understanding of molybdate behavior in aqueous solution.

### 3.5. 8-HQA as a Possible Molybdophore: Sequestering Ability Assessment

To be considered as a possible molybdophore, *8-HQA* should prove to be a good sequestering agent towards molybdate in various conditions. For the assessment of the sequestering ability of a ligand, evaluations made on the sole analysis of the stability constants of its complexes are not always sufficient, so that several “parameters” have been proposed in the past for these purposes, some of them reviewed in [29,39]. One of them, as already stated in the experimental section, is pL_0.5_, suggested by some of the authors of this work [29]. To this aim, a series of sequestration diagrams of MoO_4_^2−^ by *8-HQA* were drawn at different pH (Figure S12), and the corresponding pL_0.5_, calculated as already described, are reported in Table 3 (together with analogous values for Fe^3+^, from [2]).

As observed, pL_0.5_ (and, consequently, the sequestering ability of *8-HQA*) for molybdate are very similar to those of Fe^3+^, also supporting *8-HQA* as a good sequestering agent for MoO_4_^2−^.

**Figure 8:**
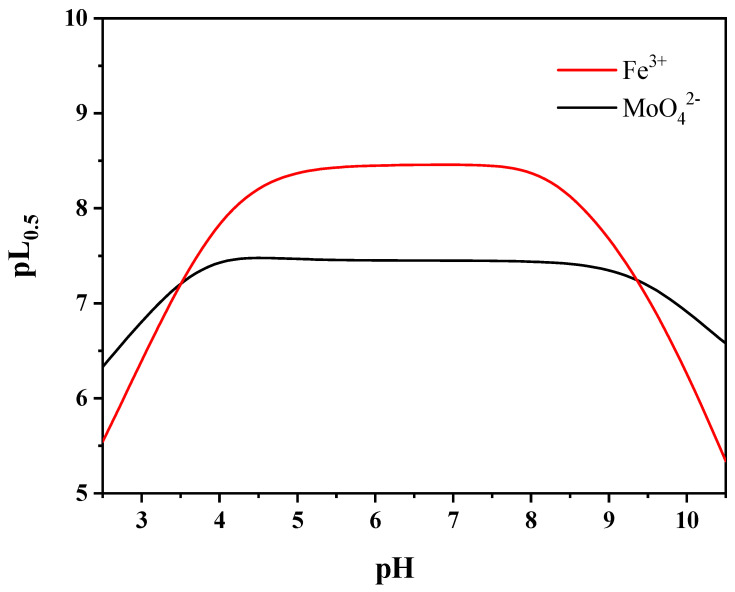
Dependence of pL_0.5_ on pH for the sequestration of Fe^3+^ (red line) and MoO_4_^2−^ (black line) by *8-HQA* in KCl_(aq)_ at *I* = 0.2 mol dm^−3^ and at *T* = 298.15 K.

Figure 8 shows the pL_0.5_ vs. pH for both MoO_4_^2−^ and Fe^3+^.

As for Fe^3+^, its pL_0.5_ tends to increase from acidic pH until it reaches a sort of plateau in the range 4.5 < pH < 9.5, to undergo a slight decrease at pH ~10.5. However, the differences in the case of molybdate are lower than one order of magnitude (pL_0.5_ is in log scale) along the whole pH range, while they are more marked in the case of Fe^3+^. This fact can be interpreted remembering that the sequestration by a ligand is a process in competition with other side-reactions. As such, while the increased pH facilitates the ligand’s deprotonation, it simultaneously favors (eventual) hydrolysis processes. So, in the case of MoO_4_^2−^, the positive (towards sequestration) effect of *8-HQA* deprotonation is “mitigated” by the concurrent molybdate deprotonation (negative effect), and no side reactions occur at very basic pH values. The mild decrease of the sequestering ability of *8-HQA* towards MoO_4_^2−^ at pH > 9.5 can be just explained by the lower relative stability of the fully deprotonated (MoO_4_)(*8-HQA*) species with respect to the monoprotonated (MoO_4_)(*8-HQA*)H. This is a further indirect indication in favor of the hypothesis of the proposed structure of complexes, in which the (MoO_4_)(*8-HQA*)H(H_2_O) species is stabilized through hydrogen bonds, which would occur to a lesser extent (or not occur) in the deprotonated species. In the case of Fe^3+^, it was already pointed out that the competition between its strong hydrolysis processes with *8-HQA* sequestration causes a significant decrease of pL_0.5_ at pH > 9.0, making the sequestering ability of *8-HQA* more pH-sensitive for Fe^3+^ than for MoO_4_^2−^.

**Figure 9:**
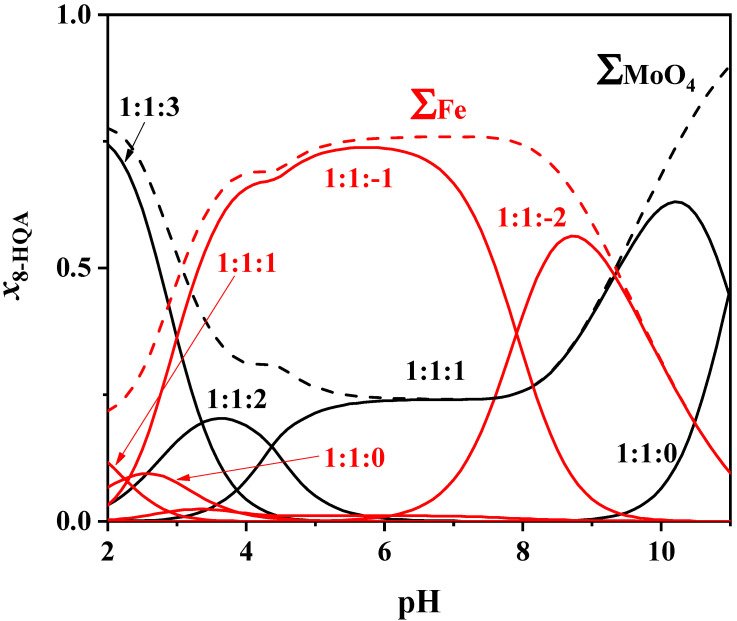
Distribution of *8-HQA* species vs. pH, in the presence of MoO_4_^2−^ (black) and Fe^3+^ (red). p:q:r indexes refer to M_p_(*8-HQA*)_q_H_r_ species, with M = MoO_4_^2−^ or Fe^3+^. Conditions: *c*_MoO4_ = *c*_Fe_ = *c_8-HQA_* = 1 × 10^−3^ mol·dm^−3^, *T* = 298.15 K and *I* = 0.2 mol·dm^−3^ in KCl_(aq)_.

As a result, pL_0.5_ of *8-HQA* towards MoO_4_^2−^ are higher or lower than those for Fe^3+^ depending on pH, suggesting that their selective sequestration by *8-HQA* may be induced by pH changes of the systems where they could be present. This could happen, for instance, along the human digestive apparatus (from stomach to different gut tracts), as it was already hypothesized in the gut of *Spodoptera* larvae between Fe^3+^ and Fe^2+^ [2]. The speciation diagram of *8-HQA* in the presence of both Fe^3+^ and MoO_4_^2−^, reported in Figure 9, supports these assumptions.

Altogether, these findings foster the hypothesis that *8-HQA* can act as a possible molybdophore.

## 4. Conclusions

The main results obtained in the present work can be summarized as follows. A series of potentiometric (ISE-H^+^, glass electrode) and UV/Vis spectrophotometric measurements were performed to define the chemical speciation of MoO_4_^2−^ in the presence of *8-HQA*, in order to evaluate the possible role of this ligand as molybdophore in real aqueous systems such as, for example, biological fluids. Based on previous findings [2] for analogous Fe^3+^/*8-HQA* and Fe^2+^/*8-HQA* systems, experiments were designed considering different concentration ratios, in order to investigate the possible formation of poly-*8-HQA* species. Nevertheless, results gave only evidence for the formation of (MoO_4_)(*8-HQA*)H_r_ species, namely (MoO_4_)(*8-HQA*)H_3_, (MoO_4_)(*8-HQA*)H_2_, (MoO_4_)(*8-HQA*)H and (MoO_4_)(*8-HQA*). Potentiometry and UV/Vis spectrophotometry, followed by computer data analysis, are the most widely used techniques, and they still remain among the most adequate and accurate for the determination of stability constants in solution. Nevertheless, the investigation of more and more complex systems (e.g., ligands with several and different binding sites, very strong chelants, unconventional conditions, multicomponent solutions, etc.) have opened up new challenges and questions for solution chemists. As such, other techniques and/or approaches do become necessary to get further information, for example, on the nature of species effectively formed, on their structure, and on their reactivity. To support the potentiometric and spectrophotometric results, further investigations were performed by means of other techniques, such as voltammetry (CV and DP-ASV), mass spectrometry and theoretical calculations, in order to confirm/rebut the speciation model proposed and to get further insights on the coordination of *8-HQA* on molybdenum. It is of major importance to underline once again here that, for a series of reasons explained in the manuscript for each set of experiments, none of these techniques, alone, can give unequivocal confirmatory answers, though all the obtained results tend towards the same hypothesis of the correctness of the proposed model. In the investigated conditions, voltammetric measurements gave evidence of the formation of only one species, along with MS results. Furthermore, no poly-*8-HQA* species were observed by MS, as well as any adduct with MoO_3_ and/or MoO_2_^2+^ moieties. On the contrary, the [(MoO_4_)(*8-HQA*)H(H_2_O)] adduct was detected an confirmed by HRMS. By means of quantomechanical calculations, it was proven how the presence of this water molecule stabilizes the configuration of the complex through hydrogen bonds, leading to a structure that is compatible with all results obtained in this work. No stable configurations were obtained for poly-*8-HQA* complexes, as well as structures with MoO_3_ and/or MoO_2_^2+^ moieties.

Worth mentioning is also the fact that, for the system investigated in this work, the use of NMR techniques (including ^17^O- and ^95^Mo-NMR) was hampered due to the relatively low *8-HQA* solubility in water (with respect to optimal concentrations for NMR), though some tests were initially performed with unsatisfactory results.

Finally, the sequestration of MoO_4_^2−^ by *8-HQA* was assessed by means of pL_0.5_ calculations at different pH, and was compared with that of Fe^3+^. *8-HQA* proved to be a good sequestering agent towards molybdate in a wide pH range, suggesting its possible role as molybdophore, especially in relation to the pH-mediated selectivity towards MoO_4_^2−^ and Fe^3+^.

## Figures and Tables

**Table 1:** Protonation constants of MoO_4_^2−^ and *8-HQA* obtained by UV/Vis spectrophotometric titrations at *T* = 298.15 K and *I* = 0.2 mol·dm^−3^ in KCl_(aq)_.

p:q:r	log *β*_pqr_^1^
1:0:1	3.97 ± 0.02
1:0:2	7.40 ± 0.02
0:1:1	9.55 ± 0.01
0:1:2	13.49 ± 0.02

^1^ log *β*_pqr_ refer to equilibrium: p MoO_4_^2−^ + q *8-HQA*^2−^ + r H^+^ = (MoO_4_)_p_(*8-HQA*)_q_H_r_^(2p+2q−r)−^; ±95% confidence interval.

**Table 2:** Stability constants of (MoO_4_)_p_(*8-HQA*)_q_H_r_^(2p+2q−r)−^ species at *T* = 298.15 K and *I* = 0.2 mol·dm^−3^ in KCl_(aq)_, by ISE-H^+^ potentiometric and spectrophotometric measurements.

p:q:r	log *β*_pqr_^1^		
	ISE-H^+^	UV/Vis	Proposed
1:1:3	24.73 ± 0.03	24.73 ± 0.02	24.73 ± 0.04
1:1:2	21.48 ± 0.02	21.26 ± 0.02	21.37 ± 0.03
1:1:1	17.00 ± 0.02	16.99 ± 0.03	17.00 ± 0.04
1:1:0	6.12 ± 0.03	5.92 ± 0.03	6.02 ± 0.05

^1^ log *β*_pqr_ refer to equilibrium: p MoO_4_^2−^ + q *8-HQA*^2−^ + r H^+^ = (MoO_4_)_p_(*8-HQA*)_q_H_r_^(2p+2q−r)−^; ±95% confidence interval.

**Table 3:** pL_0.5_ values for the sequestration of MoO_4_^2−^ and Fe^3+ 1)^ by *8-HQA* at *T* = 298.15 K and *I* = 0.2 mol·dm^−3^ in KCl_(aq)_, at different pH.

	**MoO_4_^2−^**								
**pH**	2.5	3.5	4.5	5.5	6.5	7.4	8.1	9.5	10.5
**pL_0.5_**	6.29	7.18	7.46	7.44	7.44	7.44	7.42	7.18	6.58
	**Fe^3+^**								
**pH**		3.0	4.0	5.0	6.0	7.4	8.1	9.0	10.0
**pL_0.5_**		6.29	7.77	8.33	8.41	8.42	8.31	8.31	6.24

^1^ From Reference [2].

## Supplementary Materials

The following are available online at https://www.mdpi.com/2218-273X/10/6/930/s1, Table S1: Protonation constants of MoO_4_^2−^ and *8-HQA* at *T* = 298.15 K and *I* = 0.2 mol·dm^−3^ in KCl_(aq)_; Table S2: Specific molar absorbances of (*8-HQA*)H_r_ and MoO_4_H_r_ species (with 0 ≤ r ≤ 2 for both ligands) at different wavelengths, at *T* = 298.15 K; Table S3: Specific molar absorbances of (MoO_4_)_p_(*8-HQA*)_q_H_r_ species at different wavelengths, at *T* = 298.15 K; Figure S1: Specific molar absorbances of (*8-HQA*)H_r_ and MoO_4_H_r_ species (with 0 ≤ r ≤ 2 for both ligands) at different wavelengths, at *T* = 298.15 K; Figure S2: Example of spectrophotometric titration. Conditions: *c*_MoO4_ = 1.8 × 10^−5^ mol·dm^−3^, *c_8-HQA_* = 2 × 10^−5^ mol·dm^−3^, *c*_H_ = 5 × 10^−3^ mol·dm^−3^, *T* = 298.15 K and *I* = 0.2 mol dm^−3^ in KCl_(aq)_; Figure S3: Distribution of molybdate species vs. pH, in the presence of *8-HQA*. p:q:r indexes refer to (MoO_4_)_p_(*8-HQA*)_q_H_r_^(2p+2q−r)−^. Conditions: *c*_MoO4_ = *c_8-HQA_* = 1 × 10^−3^ mol·dm^−3^, *T* = 298.15 K and *I* = 0.2 mol·dm^−3^ in KCl_(aq)_.; Figure S4: Distribution of molybdate species vs. pH, in the presence of *8-HQA*. p:q:r indexes refer to (MoO_4_)_p_(*8-HQA*)_q_H_r_^(2p+2q−r)−^. Conditions: *c*_MoO4_ = 1 × 10^−5^ mol·dm^−3^, *c_8-HQA_* = 2 × 10^−5^ mol·dm^−3^, *T* = 298.15 K and *I* = 0.2 mol·dm^−3^ in KCl_(aq)_; Figure S5: CVs of molybdate in the presence of *8-HQA*, at different pH Conditions: *c*_MoO4_ = *c_8-HQA_* = 3 × 10^−4^ mol·dm^−3^, *T* = 298.15 K and *I* = 0.2 mol·dm^−3^ in KCl_(aq)_, scan rate 100 mV/s; Figure S6: CVs of molybdate in the presence of *8-HQA* at different scan rates. Conditions: *c*_MoO4_ = *c_8-HQA_* = 3 × 10^−4^ mol·dm^−3^, *T* = 298.15 K and *I* = 0.2 mol·dm^−3^ in KCl_(aq)_, pH = 7.3; Figure S7: Superimposed ESI-MS spectra of molybdate/*8-HQA* solutions at different *c*_MoO4_:*c_8-HQA_* ratios, at pH~2.5. Black lines: *c*_MoO4_:*c_8-HQA_* = 1:1; red lines: *c*_MoO4_:*c_8-HQA_* = 1:2; blue lines: *c*_MoO4_:*c_8-HQA_* = 1:3; Figure S8: Superimposed ESI-MS spectra of molybdate/*8-HQA* solutions at different *c*_MoO4_:*c_8-HQA_* ratios, at pH~4.0. Black lines: *c*_MoO4_:*c_8-HQA_* = 1:1; red lines: *c*_MoO4_:*c_8-HQA_* = 1:2; blue lines: *c*_MoO4_:*c_8-HQA_* = 1:3; Figure S9: Superimposed ESI-MS spectra of molybdate/*8-HQA* solutions at different *c*_MoO4_:*c_8-HQA_* ratios, at pH~8.0. Black lines: *c*_MoO4_:*c_8-HQA_* = 1:1; red lines: *c*_MoO4_:*c_8-HQA_* = 1:2; blue lines: *c*_MoO4_:*c_8-HQA_* = 1:3; Figure S10: Superimposed ESI-MS spectra of molybdate/*8-HQA* solutions at different *c*_MoO4_:*c_8-HQA_* ratios, at pH ~ 11.0. Black lines: *c*_MoO4_:*c_8-HQA_* = 1:1; red lines: *c*_MoO4_:*c_8-HQA_* = 1:2; blue lines: *c*_MoO4_:*c_8-HQA_* = 1:3; Figure S11: ESI-MS spectra of molybdate/*8-HQA* solutions at different pH. Conditions: *c*_MoO4_ = *c_8-HQA_* = 5 × 10^−4^ mol·dm^−3^. (a) pH~2.5; (b) pH~4.0; (c) pH~8.0; (d) pH~11.0; Figure S12: Sequestration diagrams of MoO_4_^2−^ by *8-HQA* at *T* = 298.15 K and *I* = 0.2 mol·dm^−3^ in KCl_(aq)_, at different pH.
