# Supplementary material for: 8-Hydroxyquinoline-2-Carboxylic Acid as Possible Molybdophore: A Multi-Technique Approach to Define Its Chemical Speciation, Coordination and Sequestering Ability in Aqueous Solution
Source: Biomolecules. 2020 Jun 18;10(6):930. doi: 10.3390/biom10060930 (PMC7356571; doi:10.3390/biom10060930)
Supplement: Supplementary file 1 [file biomolecules-10-00930-s001.pdf]

biomolecules

Supplementary Material

8-Hydroxyquinoline-2-carboxylic acid as possible molybdophore: a multi-technique approach to define its chemical speciation, coordination and sequestering ability in aqueous solution.

Table S1. Protonation constants of MoO_4^{2-} [1] and *8-HQA* [2] at T = 298.15 K and I = 0.2 mol dm^{-3} in $KCl_{(aq)}$

**p:q:r**	**log β_{pqr} [3]**
1:0:1	3.97
1:0:2	7.41
7:0:8	52.42
7:0:9	57.88
7:0:10	62.10
7:0:11	65.21
0:1:1	9.56
0:1:2	13.52

[1] from ref. [30]; [2] from ref. [2]; [3] log β_{pqr} refer to equilibrium: p MoO_4^{2-} + q $8\text{-}HQA^{2-}$ + r H^+ = $(MoO_4)_p(8\text{-}HQA)_qH_r^{(2p+2q-r)-}$.

biomolecules

Table S2. Specific molar absorbances of (*8-HQA*)H_r and MoO_4H_r species (with $0 \le r \le 2$ for both ligands) at different wavelengths, at T = 298.15 K

λ [1]	ε [2]					
	8-HQA	(*8-HQA*)H	(*8-HQA*)H_2	MoO_4	MoO_4H	MoO_4H_2
205	59461	13839	28450	14500	6610	3970
206	55178	16284	27691	14700	6880	4230
207	48738	17626	25455	14600	7200	4250
208	42846	18166	23244	14200	7330	4540
209	37892	18785	20909	14200	7460	4700
210	34346	18801	19022	14200	7840	4830
211	31396	18188	17215	14100	7900	5090
212	29001	17766	15591	13400	7980	5270
213	26335	17238	13908	13100	8070	5560
214	24519	16484	12751	12800	8130	5750
215	22997	15893	11866	12500	8080	6010
216	21757	15295	11171	12200	8160	6220
217	20763	14916	10599	11700	8070	6430
218	19532	14429	9985	11300	8030	6670
219	18638	14190	9534	11100	7950	6920
220	17956	14006	9201	10700	7880	7100
221	17225	13920	8824	10400	7760	7310
222	16453	13698	8376	10100	7580	7500
223	15749	13441	7966	9760	7300	7730
224	14991	13184	7509	9570	7100	7930
225	14579	13070	7243	9460	6990	8110
226	13901	12830	6823	9240	6810	8260
227	13846	13066	6859	8990	6730	8380
228	13119	12742	6359	8760	6490	8550
229	12878	12953	6233	8560	6330	8640
230	12672	13784	6086	8400	6200	8730
231	12255	13650	5852	8190	6020	8840
232	12768	14361	6665	8030	5930	8900
233	11990	14882	6228	7860	5770	8950
234	11661	15517	6335	7760	5520	8990
235	12160	16576	6902	7530	5450	8970
236	11926	17721	7325	7410	5220	8980
237	11587	19840	7721	7230	5140	8950
238	11717	21586	8553	7110	4860	8940
239	11233	23448	9045	6960	4710	8900
240	10854	25823	9747	6800	4580	8830
241	11108	28127	11045	6720	4360	8790
242	10917	30728	12191	6670	4240	8730
243	10778	32833	13304	6560	4110	8630
244	10784	34664	14391	6490	3880	8570
245	10736	37259	16070	6360	3740	8470
246	11245	39922	18260	6310	3510	8410
247	11355	41553	19932	6240	3390	8330
248	11809	42774	21695	6190	3290	8230
249	12428	43457	23519	6190	2980	8240

250	13134	43664	25522	6100	2960	8120
251	14261	43353	27882	5890	2930	7980
252	15709	42143	30273	5990	2780	7920
253	17126	40185	32291	5840	2700	7830
254	18414	37759	33843	5820	2510	7750
255	20219	33945	35617	5700	2480	7680
256	22715	29201	37642	5740	2380	7570
257	25119	24771	39171	5630	2320	7520
258	26912	20800	39885	5530	2320	7410
259	29328	16324	40414	5340	2190	7290
260	31265	13267	40660	5220	2230	7210
261	32942	10988	40539	5110	2180	7040
262	34977	8399	39591	5070	2170	6980
263	36885	6598	38652	5010	2080	6930
264	37928	5699	37534	4920	1970	6860
265	39466	4862	35450	4710	1890	6710
266	40409	3469	33029	4540	1700	6640
267	40974	3622	30321	4300	1680	6560
268	41152	3639	27983	4110	1500	6470
269	40839	3694	25697	3900	1410	6350
270	39833	3810	22037	3710	1290	6210
271	38430	4137	18636	3560	1240	6080
272	37037	4318	16005	3390	1110	5980
273	35048	4416	13472	3150	973	5810
274	33171	4626	11606	2870	741	5720
275	30665	4755	9713	2710	714	5530
276	27026	5112	7708	2570	644	5370
277	23853	5038	6438	2400	561	5200
278	21547	5057	5746	2290	477	5060
279	18675	4968	4960	2200	435	4930
280	15927	4775	4372	2070	439	4770
281	13016	4777	3897	1970	394	4600
282	10848	4681	3631	1860	349	4450
283	9628	4698	3434	1780	305	4310
284	8213	4516	3249	1690	291	4190
285	7163	4553	3159	1580	321	4030
286	6185	4451	3039	1480	305	3820
287	5535	4341	2948	1370	333	3570
288	5219	4291	2911	1300	343	3350
289	4942	4297	2929	1200	342	3190
290	4595	4191	2833	1130	384	2960
291	4374	4188	2795	1040	377	2800
292	4223	4140	2834	993	390	2660
293	4141	4129	2861	931	357	2530
294	3964	4052	2783	893	339	2450
295	3946	4070	2873	856	359	2350
296	3950	4079	2936	806	340	2280
297	3896	4099	2990	757	316	2180
298	3821	3995	2966	738	315	2110
299	3898	4091	3099	724	308	2080
300	3784	3895	3078	675	306	1980
301	3806	3822	3125	669	271	1930

302	3877	3953	3239	659	244	1900
303	3816	3941	3240	636	232	1860
304	3773	4097	3285	603	239	1810
305	3669	3979	3194	571	253	1760
306	3720	3997	3286	537	268	1700
307	3777	3891	3382	518	273	1650
308	3670	3805	3357	505	260	1610
309	3682	3786	3403	495	244	1610
310	3616	3710	3400	481	251	1560
311	3726	3745	3602	453	249	1530
312	3689	3651	3624	437	223	1520
313	3697	3699	3715	426	222	1480
314	3783	3618	3844	418	200	1490
315	3775	3632	3933	421	198	1440
316	3690	3323	3863	391	209	1420
317	3786	3452	3939	385	192	1370
318	4026	3596	4115	387	208	1320
319	3897	3428	3951	377	181	1330
320	3826	3371	3875	358	184	1310
321	3835	3271	3807	361	161	1270
322	3784	3072	3708	356	146	1250
323	3762	3203	3749	341	149	1220
324	3905	3033	3861	370	117	1200
325	3888	3138	3895	350	130	1170
326	3875	3107	3936	327	139	1140
327	3983	2974	4061	324	146	1080
328	3829	2680	3932	324	138	1070
329	3816	2730	3904	332	128	1030
330	3997	2824	3976	338	132	994
331	4004	2757	3799	334	117	953
332	4046	2942	3637	314	116	893
333	4026	2793	3205	316	133	899
334	4027	2753	2971	313	131	886
335	4034	2704	2701	303	138	841
336	3984	2727	2403	309	128	805
337	4215	2932	2490	296	131	777
338	4268	3006	2431	285	148	729
339	4230	2821	2315	317	127	731
340	3975	2451	2002	309	114	706
341	4060	2633	2067	302	134	682
342	4137	2680	2115	300	112	656
343	4072	2533	2044	304	109	627
344	4293	2789	2276	308	107	616
345	4094	2669	2134	307	104	602
346	3963	2505	2048	314	100	589
347	4110	2589	2202	305	111	557
348	4014	2578	2164	308	108	547
349	3934	2424	2110	291	107	532
350	3998	2329	2178	301	96	515
351	4033	2387	2232	323	71	493
352	4009	2404	2201	289	93	486
353	4090	2253	2267	283	83	445

354	4099	2193	2285	310	72	472
355	4007	2141	2225	307	69	448
356	4143	2203	2346	291	70	430
357	4129	2097	2292	299	72	413
358	4160	2163	2329	298	43	424
359	3985	1911	2127	291	40	406
360	4022	1930	2141	292	28	413
361	4179	1867	2255	0	0	0
362	4257	1823	2281	0	0	0
363	4170	1722	2215	0	0	0
364	4232	1625	2209	0	0	0
365	4362	1618	2293	0	0	0
366	4306	1546	2268	0	0	0
367	4395	1558	2343	0	0	0
368	4320	1474	2220	0	0	0
369	4614	1634	2468	0	0	0
370	4403	1348	2245	0	0	0
371	4500	1276	2272	0	0	0
372	4607	1310	2337	0	0	0
373	4578	1206	2276	0	0	0
374	4585	1165	2246	0	0	0
375	4598	1110	2233	0	0	0
376	4679	1119	2247	0	0	0
377	4704	1060	2235	0	0	0
378	4702	957	2215	0	0	0
379	4719	954	2217	0	0	0
380	4755	969	2219	0	0	0
381	4741	915	2156	0	0	0
382	4784	994	2144	0	0	0
383	4879	949	2180	0	0	0
384	4846	869	2147	0	0	0
385	4885	882	2157	0	0	0
386	4844	845	2103	0	0	0
387	4857	767	2081	0	0	0
388	4951	794	2067	0	0	0
389	4996	864	2089	0	0	0
390	4952	879	2055	0	0	0
391	4913	930	2030	0	0	0
392	4921	927	2004	0	0	0
393	4948	913	1998	0	0	0
394	4954	827	1973	0	0	0
395	4934	845	1963	0	0	0
396	4948	859	1956	0	0	0
397	4914	751	1907	0	0	0
398	4898	737	1881	0	0	0
399	4902	756	1863	0	0	0
400	4848	831	1845	0	0	0
401	4858	799	1830	0	0	0
402	4813	785	1805	0	0	0
403	4812	770	1774	0	0	0
404	4811	746	1749	0	0	0
405	4812	759	1755	0	0	0

406	4752	795	1743	0	0	0
407	4713	815	1703	0	0	0
408	4703	769	1694	0	0	0
409	4618	793	1643	0	0	0
410	4617	769	1640	0	0	0
411	4589	775	1623	0	0	0
412	4511	815	1595	0	0	0
413	4491	795	1584	0	0	0
414	4464	802	1564	0	0	0
415	4392	764	1495	0	0	0
416	4387	830	1511	0	0	0
417	4331	838	1447	0	0	0
418	4314	846	1461	0	0	0
419	4233	867	1419	0	0	0
420	4166	834	1396	0	0	0
421	4154	840	1382	0	0	0
422	4080	863	1353	0	0	0
423	4063	816	1356	0	0	0
424	4088	825	1392	0	0	0
425	3957	782	1309	0	0	0
426	3918	822	1306	0	0	0
427	3879	867	1269	0	0	0
428	3847	863	1265	0	0	0
429	3810	903	1280	0	0	0
430	3728	910	1218	0	0	0
431	3717	960	1223	0	0	0
432	3662	912	1188	0	0	0
433	3582	876	1143	0	0	0
434	3563	886	1148	0	0	0
435	3516	894	1124	0	0	0
436	3471	860	1127	0	0	0
437	3449	894	1160	0	0	0
438	3383	939	1137	0	0	0
439	3317	938	1098	0	0	0
440	3293	907	1068	0	0	0
441	3246	851	1030	0	0	0
442	3235	905	1053	0	0	0
443	3196	897	1034	0	0	0
444	3166	837	1036	0	0	0
445	3117	810	1020	0	0	0
446	3136	911	1048	0	0	0
447	3066	896	997	0	0	0
448	2992	845	983	0	0	0
449	2958	955	992	0	0	0
450	2935	873	986	0	0	0
451	2895	923	974	0	0	0
452	2862	939	970	0	0	0
453	2872	1021	952	0	0	0
454	2825	1011	922	0	0	0
455	2814	883	934	0	0	0
456	2756	923	929	0	0	0
457	2760	915	945	0	0	0

458	2698	928	895	0	0	0
459	2692	980	918	0	0	0
460	2676	974	915	0	0	0
461	2685	911	922	0	0	0
462	2686	883	911	0	0	0
463	2607	937	859	0	0	0
464	2606	1021	866	0	0	0
465	2616	982	896	0	0	0
466	2603	861	899	0	0	0
467	2543	848	881	0	0	0
468	2591	899	892	0	0	0
469	2579	894	871	0	0	0
470	2587	1022	897	0	0	0
471	2542	986	871	0	0	0
472	2530	1020	885	0	0	0
473	2491	940	868	0	0	0
474	2504	937	850	0	0	0
475	2491	962	833	0	0	0
476	2514	1048	869	0	0	0
477	2512	1021	867	0	0	0
478	2369	1004	795	0	0	0
479	2423	1029	850	0	0	0
480	2402	959	838	0	0	0
481	2414	944	833	0	0	0
482	2438	901	836	0	0	0
483	2419	878	839	0	0	0
484	2414	926	839	0	0	0
485	2390	886	827	0	0	0
486	2401	869	838	0	0	0
487	2435	895	840	0	0	0
488	2446	822	825	0	0	0
489	2477	852	857	0	0	0
490	2400	848	848	0	0	0
491	2372	878	835	0	0	0
492	2375	900	835	0	0	0
493	2351	930	814	0	0	0
494	2384	1003	852	0	0	0
495	2378	967	845	0	0	0

[1] in nm; [2] in $(mol\ dm^{-3})^{-1}\ cm^{-1}$.

Table S3. Specific molar absorbances of $(MoO_4)_p(8\text{-}HQA)_qH_r$ species at different wavelengths, at T = 298.15 K

λ [1]	ε [2]			
	**$(MoO_4)(8\text{-}HQA)H_3$**	**$(MoO_4)(8\text{-}HQA)H_2$**	**$(MoO_4)(8\text{-}HQA)H$**	**$(MoO_4)(8\text{-}HQA)H$**
205	20523	34492	17458	111100
206	20849	34906	16742	110260
207	20524	33415	17036	99768
208	20222	31536	17884	85377
209	19677	28860	18614	72899
210	18748	26575	18870	63420
211	17767	24633	19040	53292
212	16685	22665	18545	45187
213	16038	20803	18181	39030
214	15576	19469	17865	34648
215	15069	18567	17355	31322
216	14628	17659	16816	27800
217	14201	16942	16201	25208
218	13855	16304	15762	22930
219	13558	15762	15361	21349
220	13202	15458	14931	19944
221	12744	14909	14458	18611
222	12258	14487	14006	17479
223	11823	14139	13710	16647
224	11452	13860	13467	15973
225	11013	13603	13131	15328
226	10590	13297	12885	14785
227	10229	13132	12675	14290
228	9922	12912	12547	13890
229	9687	12867	12458	13648
230	9824	13364	12664	13446
231	9648	13252	12663	13475
232	9261	12934	12342	12703
233	9573	13366	12971	12823
234	9452	13666	13250	11987
235	9484	14461	13498	11673
236	9826	15779	14003	11072
237	9794	15816	14433	10141
238	9852	16763	15159	8970
239	10492	18521	15929	9074
240	11177	19089	17059	8166
241	11591	20562	17655	7029
242	12299	21581	18683	6125
243	12884	22836	19401	5513
244	13697	24364	20208	4916
245	14886	26089	21691	4077
246	16351	27821	22978	3401
247	17934	29583	23903	3193
248	18791	30444	24606	3072
249	20087	31660	25112	3093

250	21816	32528	26389	3260
251	23795	32962	26407	4346
252	25814	33320	27136	5285
253	27064	33650	26842	6317
254	28444	33048	26545	8076
255	30564	31835	25835	10733
256	31764	31833	25074	13208
257	32856	29038	23725	15929
258	33686	27941	22868	18300
259	33843	27122	21800	20377
260	34100	24979	20775	23685
261	33946	24035	19814	26361
262	33302	22498	19198	28655
263	32470	20860	18693	30273
264	31229	20206	18111	31872
265	29847	18689	17458	33446
266	27631	17368	16667	34889
267	25373	16431	15831	35046
268	23891	15543	15478	34889
269	21433	14138	14667	34224
270	18855	13021	13901	33489
271	15253	11579	12481	31158
272	12977	10583	11519	29147
273	11628	10027	10951	27867
274	9761	8968	9984	25331
275	7900	8025	9020	22014
276	6193	7231	7916	18554
277	5371	6491	7151	16158
278	4608	6083	6445	13931
279	4090	5696	5904	11846
280	3607	5349	5179	9474
281	3126	4939	4480	7124
282	2904	4745	4005	5686
283	2792	4678	3683	4619
284	2651	4481	3384	3724
285	2520	4326	3006	2667
286	2418	4090	2744	1769
287	2447	4082	2618	1455
288	2433	4049	2442	1198
289	2341	3902	2290	794
290	2428	3969	2323	597
291	2366	3799	2181	335
292	2402	3830	2215	256
293	2379	3796	2180	101
294	2455	3776	2237	94
295	2388	3696	2119	0
296	2437	3702	2089	0
297	2504	3689	2133	0
298	2531	3726	2152	0
299	2537	3679	2102	0
300	2603	3672	2144	0
301	2705	3702	2226	0

302	2696	3707	2173	0
303	2747	3739	2214	0
304	2796	3768	2215	0
305	2853	3761	2234	0
306	2894	3666	2287	0
307	2919	3885	2263	0
308	2935	3865	2218	0
309	2992	3904	2239	0
310	3056	3791	2227	0
311	3109	3714	2248	0
312	3150	3836	2174	0
313	3204	3659	2118	0
314	3230	3802	2193	0
315	3296	3729	2173	0
316	3352	3768	2235	0
317	3301	3653	2137	0
318	3224	3568	2048	0
319	3202	3526	2082	0
320	3151	3602	2075	0
321	3150	3565	2052	0
322	3081	3493	2044	0
323	3159	3460	2090	0
324	3110	3411	1931	0
325	3099	3368	1914	0
326	3198	3438	1977	0
327	3191	3412	1908	0
328	3228	3345	1902	0
329	3369	3226	1984	200
330	3177	3278	1921	103
331	3034	3272	1868	204
332	2924	3248	1906	368
333	2697	3164	1822	433
334	2432	3072	1731	572
335	2277	2950	1833	692
336	2097	2772	1712	807
337	1855	2802	1585	752
338	1823	2763	1598	835
339	1723	2625	1507	898
340	1791	2631	1641	1049
341	1708	2520	1569	1024
342	1601	2407	1417	848
343	1663	2491	1522	993
344	1517	2279	1366	684
345	1638	2440	1495	929
346	1621	2460	1474	887
347	1590	2369	1423	727
348	1644	2438	1402	876
349	1656	2302	1415	821
350	1561	2289	1246	733
351	1558	2281	1317	627
352	1618	2285	1294	722
353	1552	2092	1146	657

354	1499	2173	1120	647
355	1610	2247	1178	772
356	1579	2159	1196	702
357	1606	2147	1165	781
358	1593	2080	1114	765
359	1636	2036	1131	821
360	1626	2009	1114	904
361	1659	2043	1077	1048
362	1603	1894	1027	933
363	1686	1943	1096	1106
364	1674	1913	1048	1135
365	1648	1853	1010	1152
366	1622	1769	1013	1150
367	1599	1844	972	1263
368	1638	1762	1055	1280
369	1498	1617	849	1184
370	1628	1702	982	1408
371	1538	1630	918	1329
372	1450	1677	801	1334
373	1579	1663	916	1452
374	1598	1693	908	1541
375	1581	1653	914	1530
376	1560	1673	886	1572
377	1558	1596	861	1615
378	1559	1580	869	1655
379	1527	1587	853	1657
380	1491	1543	829	1651
381	1467	1525	794	1670
382	1464	1441	780	1677
383	1449	1431	779	1654
384	1444	1441	778	1705
385	1429	1453	789	1705
386	1428	1481	819	1752
387	1413	1484	809	1766
388	1402	1446	795	1715
389	1354	1441	745	1675
390	1363	1423	732	1760
391	1329	1378	716	1752
392	1324	1362	727	1769
393	1309	1362	739	1724
394	1277	1345	727	1679
395	1258	1371	718	1693
396	1227	1341	694	1639
397	1206	1341	691	1621
398	1207	1347	702	1621
399	1195	1288	689	1585
400	1188	1301	689	1608
401	1162	1295	671	1566
402	1140	1290	657	1547
403	1136	1260	659	1490
404	1112	1244	634	1451
405	1094	1258	640	1409

406	1056	1231	620	1367
407	1039	1260	603	1331
408	1034	1249	604	1311
409	985	1235	571	1274
410	964	1239	557	1235
411	950	1218	556	1175
412	938	1195	551	1120
413	902	1193	522	1054
414	930	1176	544	1042
415	919	1160	523	1017
416	878	1134	505	934
417	857	1156	478	890
418	810	1142	438	810
419	798	1108	431	765
420	791	1094	449	714
421	761	1077	408	652
422	748	1078	409	601
423	714	1096	389	542
424	691	1112	363	468
425	713	1108	395	476
426	678	1108	378	401
427	701	1151	376	422
428	669	1110	351	320
429	650	1111	345	268
430	644	1116	315	226
431	624	1121	293	180
432	629	1114	311	146
433	626	1079	309	98
434	594	1027	276	17
435	596	1040	273	6
436	585	1051	280	0
437	541	1050	245	0
438	533	1050	239	0
439	527	1019	217	0
440	510	1040	194	0
441	502	1010	173	0
442	481	1003	163	0
443	488	1002	166	0
444	476	990	163	0
445	463	995	165	0
446	453	991	151	0
447	445	947	122	0
448	419	950	109	0
449	403	936	87	0
450	389	918	74	0
451	393	882	77	0
452	402	846	77	0
453	391	822	44	0
454	411	837	62	0
455	379	870	42	0
456	389	898	58	0
457	358	917	43	0

458	390	938	62	0
459	363	904	39	0
460	362	867	34	0
461	349	878	25	0
462	347	864	10	0
463	370	869	36	0
464	359	858	19	0
465	362	861	19	0
466	368	862	30	0
467	400	897	53	0
468	385	901	21	0
469	385	881	8	0
470	371	896	0	0
471	370	873	0	0
472	369	885	0	0
473	371	909	0	0
474	385	882	0	0
475	364	847	0	0
476	344	839	0	0
477	334	849	0	0
478	357	846	0	0
479	334	840	0	0
480	354	884	0	0
481	332	864	0	0
482	304	859	0	0
483	317	881	0	0
484	337	893	0	0
485	346	916	0	0
486	332	908	0	0
487	332	907	0	0
488	330	916	0	0
489	335	971	0	0
490	324	930	0	0
491	333	973	0	0
492	328	1002	0	0
493	336	1041	0	0
494	327	991	0	0
495	316	929	0	0

19 [1] in nm; [2] in $(mol\ dm^{-3})^{-1}\ cm^{-1}$.

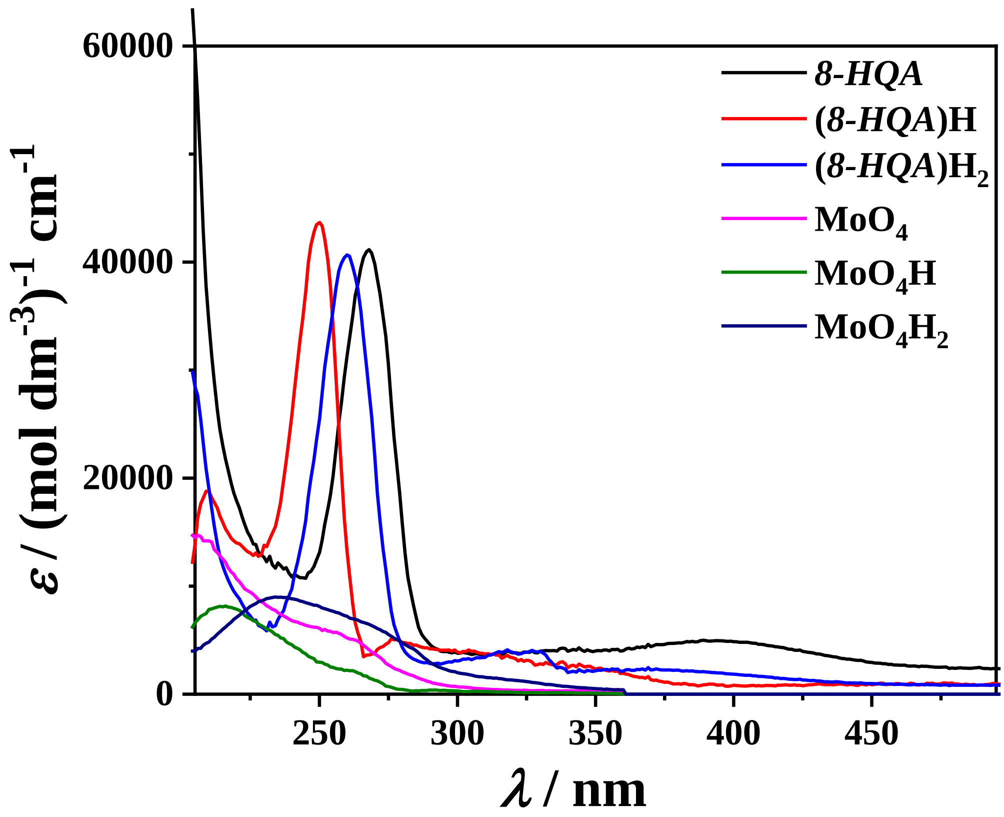

Figure S1. Specific molar absorbances of (*8-HQA*)H_r and MoO_4H_r species (with $0 \le r \le 2$ for both ligands) at different wavelengths, at T = 298.15 K.

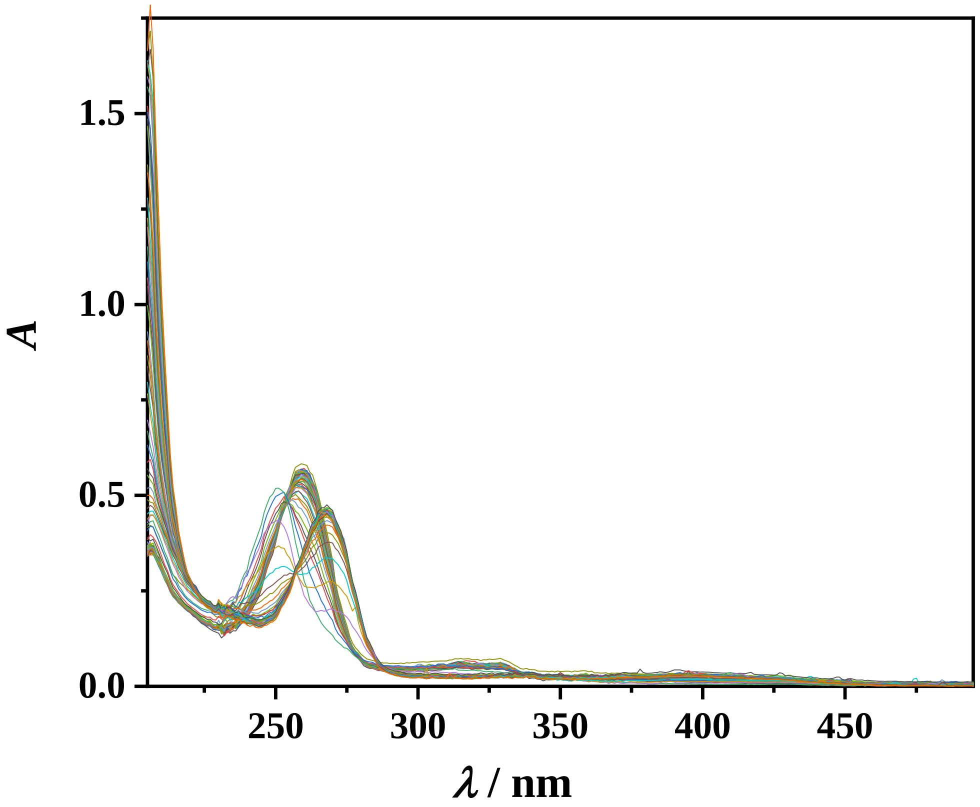

Figure S2. Example of spectrophotometric titration. Conditions: c_{MoO4} = $1.8\cdot10^{-5}$ mol·dm^{-3}, $c_{8\text{-}HQA}$ = $2\cdot10^{-5}$ mol·dm^{-3}, c_{H} = $5\cdot10^{-3}$ mol·dm^{-3}, T = 298.15 K and I = 0.2 mol dm^{-3} in $KCl_{(aq)}$.

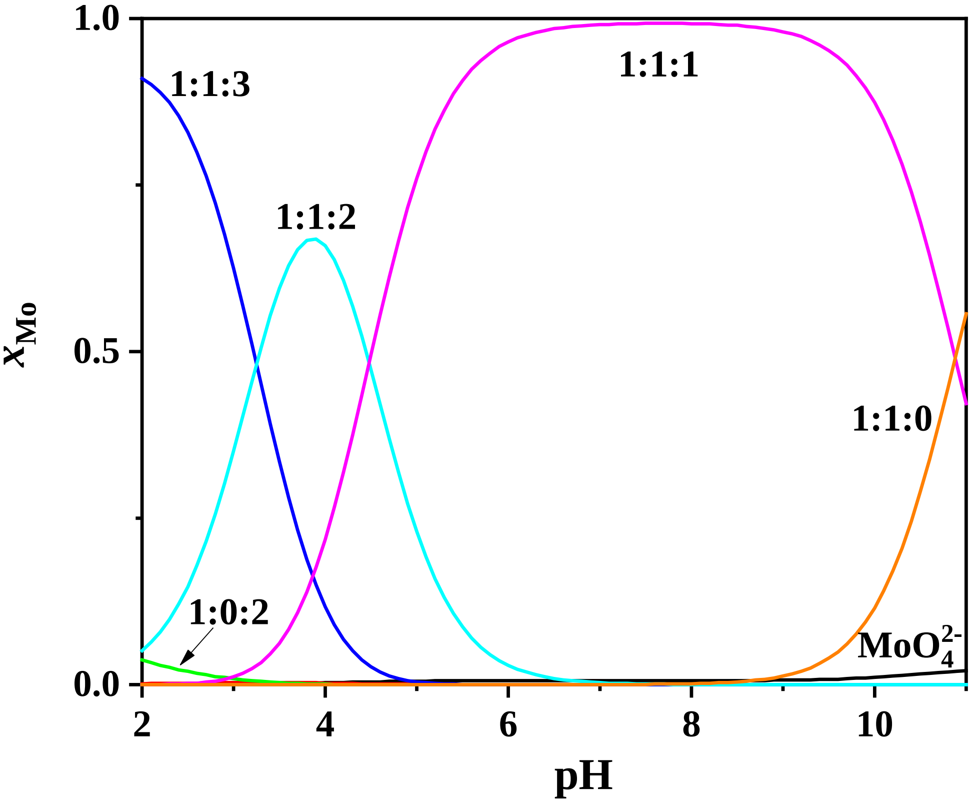

Figure S3. Distribution of molybdate species *vs.* pH, in the presence of *8-HQA*. p:q:r indexes refer to $(MoO_4)_p(8\text{-}HQA)_qH_r^{(2p+2q-r)-}$. Conditions: $c_{MoO4} = c_{8\text{-}HQA} = 1 \cdot 10^{-3}$ mol·dm^{-3}, T = 298.15 K and I = 0.2 mol dm^{-3} in $KCl_{(aq)}$.

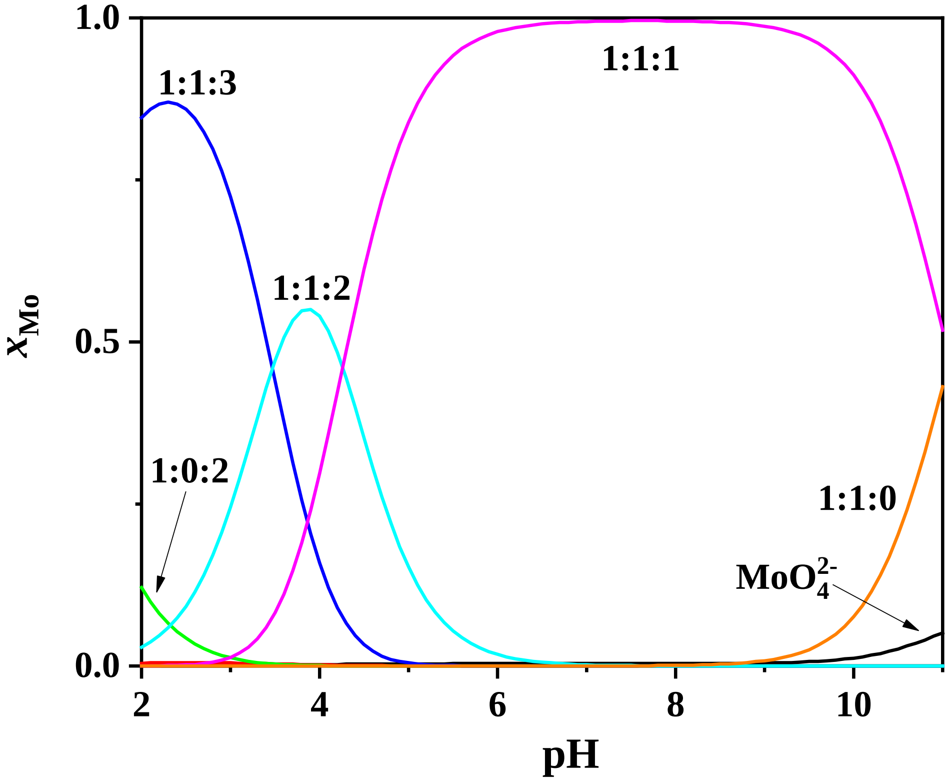

Figure S4. Distribution of molybdate species *vs.* pH, in the presence of *8-HQA*. p:q:r indexes refer to $(MoO_4)_p(8\text{-}HQA)_qH_r^{(2p+2q-r)-}$. Conditions: c_{MoO4} = 1·10^{-5} mol·dm^{-3}, $c_{8\text{-}HQA}$ = 2·10^{-5} mol·dm^{-3}, *T* = 298.15 K and *I* = 0.2 mol dm^{-3} in $KCl_{(aq)}$.

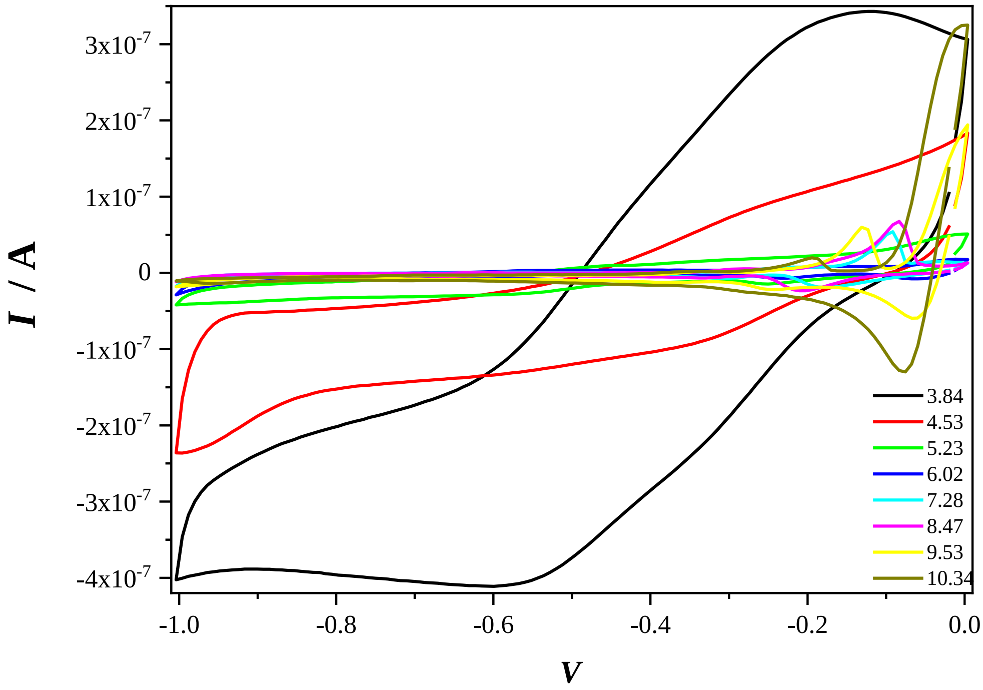

Figure S5. CVs of molybdate in the presence of *8-HQA*, at different pH Conditions: c_{MoO4} = $c_{8\text{-}HQA}$ = $3 \cdot 10^{-4}$ mol dm^{-3}, T = 298.15 K and I = 0.2 mol dm^{-3} in $KCl_{(aq)}$, scan rate 100 mV/s.

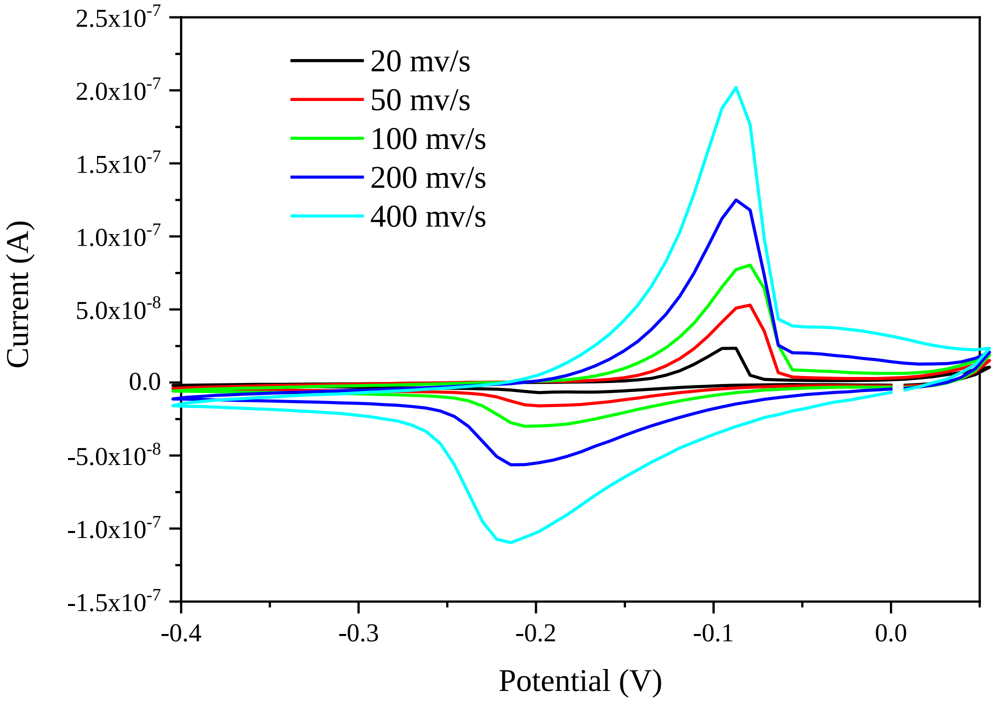

Figure S6. CVs of molybdate in the presence of *8-HQA* at different scan rates. Conditions: c_{MoO4} = $c_{8\text{-}HQA}$ = $3 \cdot 10^{-4}$ mol dm^{-3}, T = 298.15 K and I = 0.2 mol dm^{-3} in $KCl_{(aq)}$, pH = 7.3.

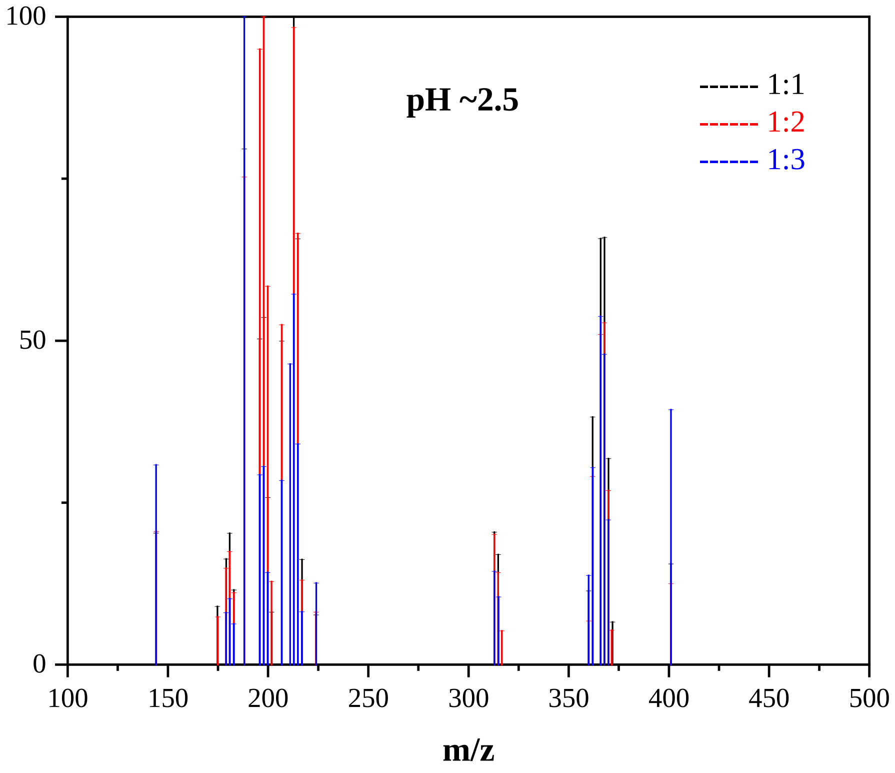

Figure S7. Superimposed ESI-MS spectra of molybdate / *8-HQA* solutions at different c_{MoO4} : $c_{8\text{-}HQA}$ ratios, at pH ~ 2.5. Black lines: c_{MoO4} : $c_{8\text{-}HQA}$ = 1:1; red lines: c_{MoO4} : $c_{8\text{-}HQA}$ = 1:2; blue lines: c_{MoO4} : $c_{8\text{-}HQA}$ = 1:3.

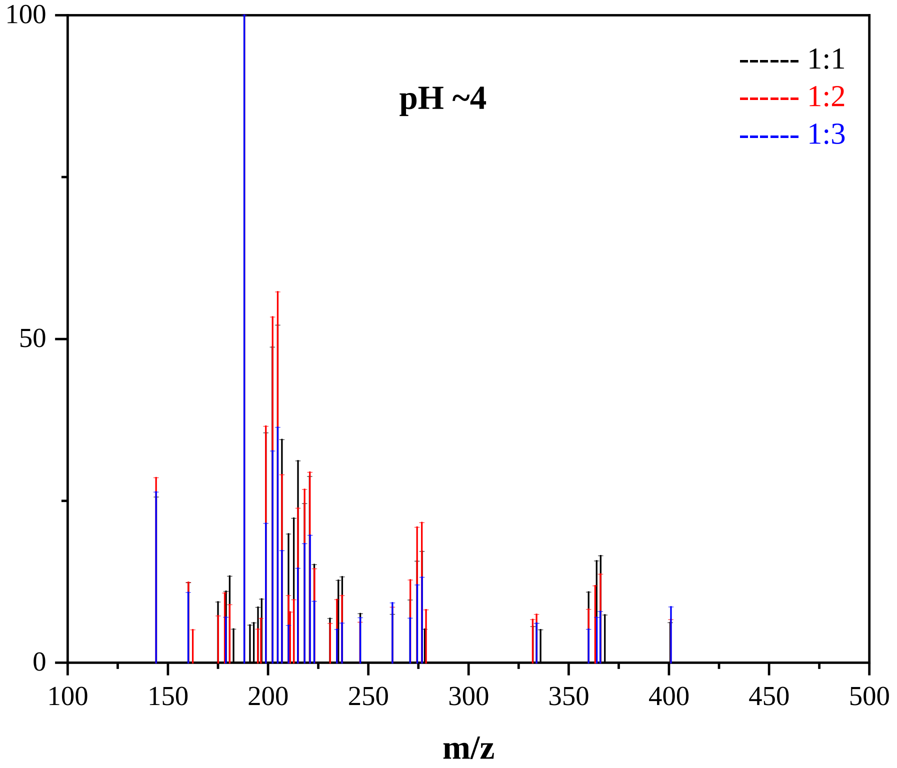

Figure S8. Superimposed ESI-MS spectra of molybdate / *8-HQA* solutions at different c_{MoO4} : $c_{8\text{-}HQA}$ ratios, at pH ~ 4.0. Black lines: c_{MoO4} : $c_{8\text{-}HQA}$ = 1:1; red lines: c_{MoO4} : $c_{8\text{-}HQA}$ = 1:2; blue lines: c_{MoO4} : $c_{8\text{-}HQA}$ = 1:3.

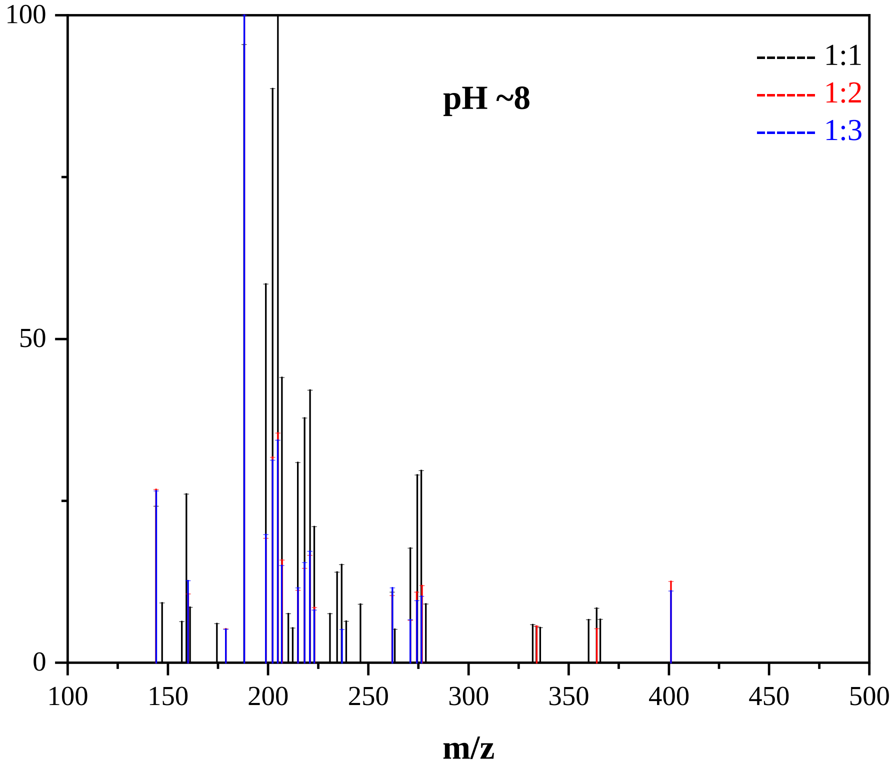

Figure S9. Superimposed ESI-MS spectra of molybdate / *8-HQA* solutions at different c_{MoO4} : $c_{8\text{-}HQA}$ ratios, at pH ~ 8.0. Black lines: c_{MoO4} : $c_{8\text{-}HQA}$ = 1:1; red lines: c_{MoO4} : $c_{8\text{-}HQA}$ = 1:2; blue lines: c_{MoO4} : $c_{8\text{-}HQA}$ = 1:3.

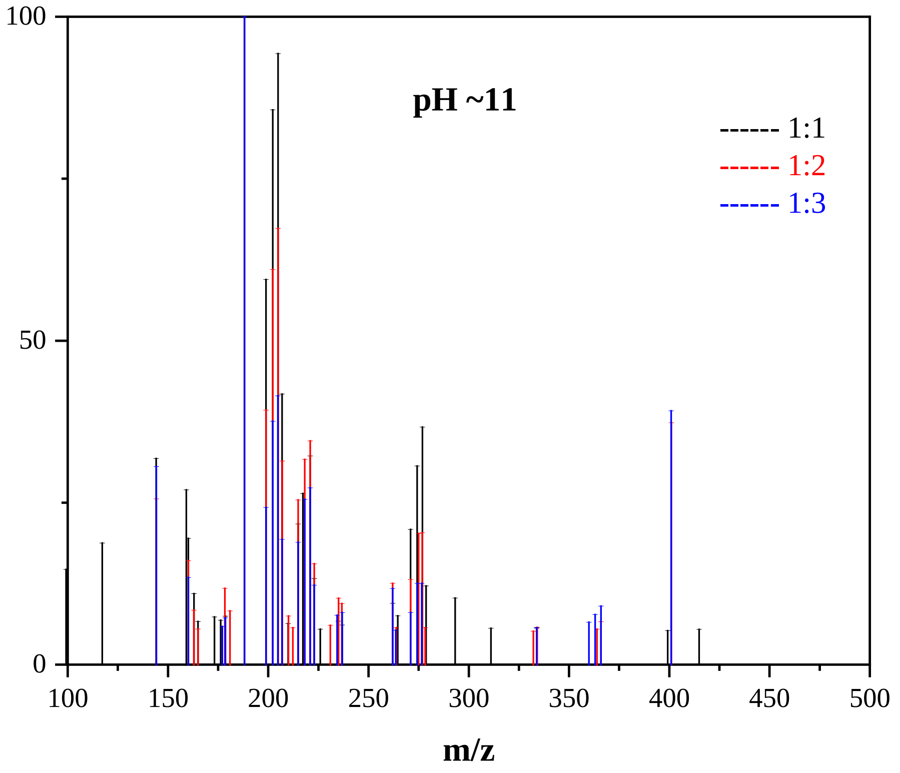

Figure S10. Superimposed ESI-MS spectra of molybdate / *8-HQA* solutions at different c_{MoO4} : $c_{8\text{-}HQA}$ ratios, at pH ~ 11.0. Black lines: c_{MoO4} : $c_{8\text{-}HQA}$ = 1:1; red lines: c_{MoO4} : $c_{8\text{-}HQA}$ = 1:2; blue lines: c_{MoO4} : $c_{8\text{-}HQA}$ = 1:3.

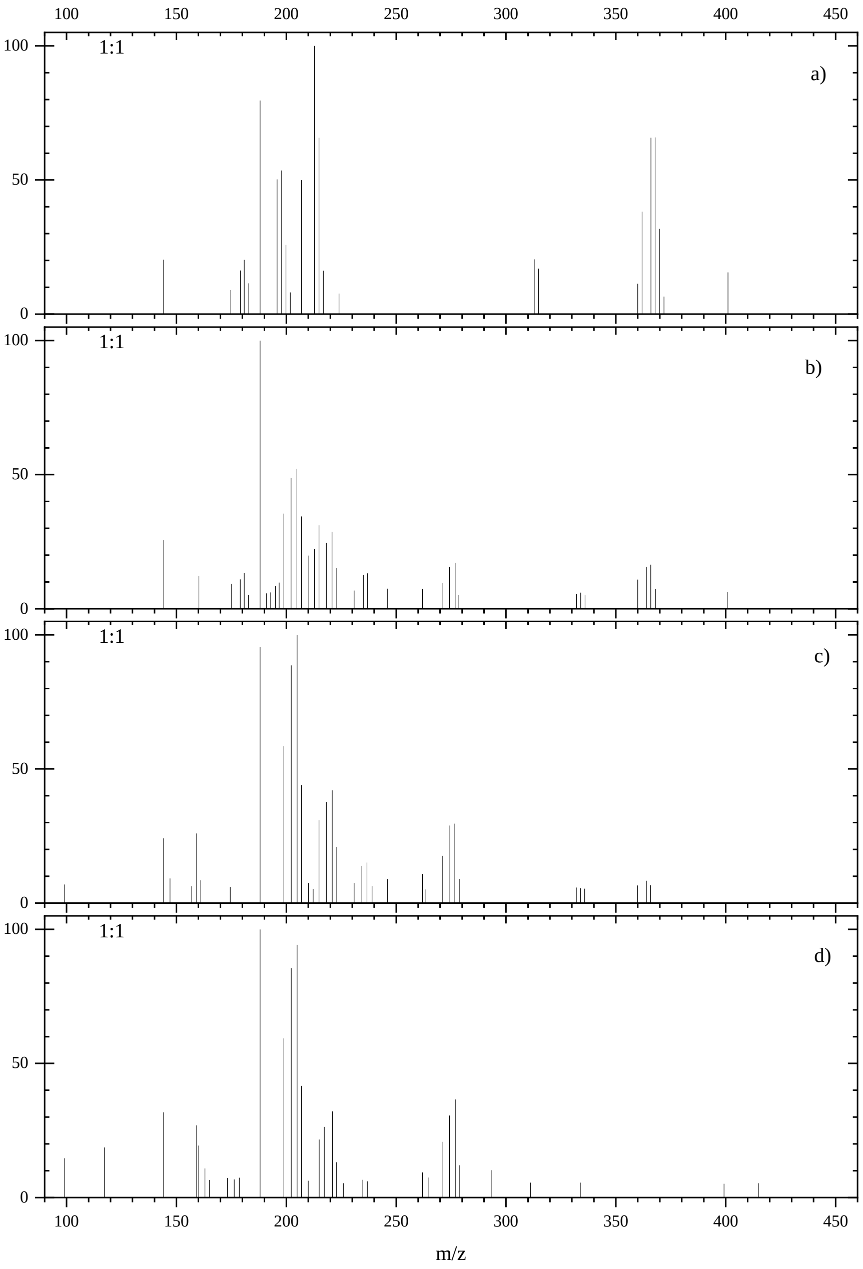

Figure S11. ESI-MS spectra of molybdate / *8-HQA* solutions at different pH. Conditions: $c_{MoO4} = c_{8\text{-}HQA} = 5 \cdot 10^{-4}$ mol dm^{-3}. a) pH ~ 2.5; b) pH ~ 4.0; c) pH ~ 8.0; d) pH ~ 11.0.

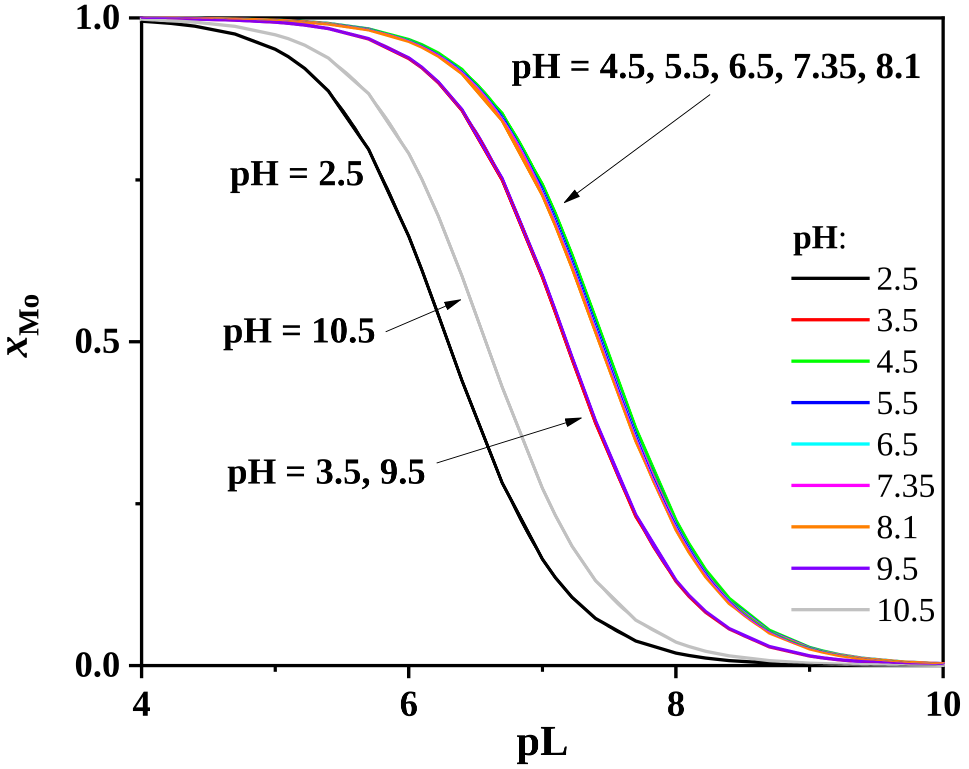

Figure S12. Sequestration diagrams of MoO_4^{2-} by *8-HQA* at T = 298.15 K and I = 0.2 mol·dm^{-3} in $KCl_{(aq)}$, at different pH.
